# The rice *zebra3* (*z3*) mutation disrupts citrate distribution and produces transverse dark-green/green variegation in mature leaves

**DOI:** 10.1186/s12284-017-0196-8

**Published:** 2018-01-05

**Authors:** Suk-Hwan Kim, Choon-Tak Kwon, Giha Song, Hee-Jong Koh, Gynheung An, Nam-Chon Paek

**Affiliations:** 1 Department of Plant Science, Plant Genomics and Breeding Institute, Research Institute of Agriculture and Life Sciences, Seoul National University, Seoul, 08826 Republic of Korea; 2 Crop Biotech Institute and Graduate School of Biotechnology, Kyung Hee University, Yongin, 17104 Republic of Korea; 3 Crop Biotechnology Institute, Institutes of Green Bio Science and Technology, Seoul National University, Pyeongchang, 25354 Republic of Korea; 4 Present address: Cold Spring Harbor Laboratory, Cold Spring Harbor, NY, 11724 USA

**Keywords:** Rice, *ZEBRA3* (*Z3*), Leaf variegation, CitMHS family, Citrate transporter

## Abstract

**Background:**

Rice *zebra* mutants are leaf variegation mutants that exhibit transverse sectors of green/yellow or green/white in developing or mature leaves. In most cases, leaf variegation is caused by defects in chloroplast biogenesis pathways, leading to an accumulation of reactive oxygen species in a transverse pattern in the leaves. Here, we examine a new type of leaf variegation mutant in rice, *zebra3* (*z3*), which exhibits transverse dark-green/green sectors in mature leaves and lacks the typical yellow or white sectors.

**Results:**

Map-based cloning revealed that the *Z3* locus encodes a putative citrate transporter that belongs to the citrate-metal hydrogen symport (CitMHS) family. CitMHS family members have been extensively studied in bacteria and function as secondary transporters that can transport metal-citrate complexes, but whether CitMHS family transporters exist in eukaryotes remains unknown. To investigate whether Z3 acts as a citrate transporter in rice, we measured citrate levels in wild-type leaves and in the dark-green and green sectors of the leaves of *z3* mutants. The results showed that citrates accumulated to high levels in the dark-green sectors of *z3* mutant leaves, but not in the green sectors as compared with the wild-type leaves.

**Conclusions:**

These results suggest that leaf variegation in the *z3* mutant is caused by an unbalanced accumulation of citrate in a transverse pattern in the leaves. Taking these results together, we propose that Z3 plays an important role in citrate transport and distribution during leaf development and is a possible candidate for a CitMHS family member in plants.

**Electronic supplementary material:**

The online version of this article (10.1186/s12284-017-0196-8) contains supplementary material, which is available to authorized users.

## Background

Citrate (citric acid) is a weak organic acid found in nature, and ubiquitously acts as an energy intermediate, solubilizing agent, carbon source, and chelator (Sarantinopoulos et al. 2003). The roles of citrate are mostly related to cellular metabolism. For instance, citrate acts as a metabolite in the Krebs cycle under aerobic conditions (Krebs and Johnson 1937), and as a source of acetyl-CoA for fatty acid synthesis (Fritsch and Beevers 1979; Matto and Modi 1970; Nelson and Rinne 1975). In plant tissues, citrate also supplies carbon skeletons for NH_4_^+^ assimilation via the glutamine synthetase (GS) and glutamate synthase (GOGAT) pathway (Chen and Gadal 1990; Agata 2008). In plants, citrate can enter into the glyoxylate cycle, allowing the plant to use two-carbon acetate as a carbon source via gluconeogenesis (Tanner and Beevers 1965).

Citrate performs other roles when transported outside the cell. Because citrate is a tricarboxylic acid with good chelating ability for cations, such as manganese, magnesium, iron, and calcium (Glusker 1980; Milewska 1988), it can chelate these metal ions and thus change their physicochemical properties. The roles of citrate in iron translocation and aluminum detoxification have been extensively studied in plants. For example, citrate is needed to solubilize inorganic iron for long-distance transport via the xylem because free ionic forms of iron are toxic and subject to precipitation in the slightly acidic xylem sap (Rellán-Álvarez et al. 2010; Green and Rogers 2004; Tiffin 1970). In many plant species, citrate is excreted into the rhizosphere, where it chelates aluminum, thereby excluding it from entering the plant (Fuente et al. 1997; Miyasaka et al. 1991; Magalhaes et al. 2007). In addition, under phosphorus-limiting conditions in *Lupinus albus*, *Brassica napus*, and the Proteaceae family of plants, the increased excretion of organic acids, particularly citrate, may enhance phosphorus uptake by displacing the phosphorus from insoluble complexes in the soil (Dinkelaker et al. 1989; Dinkelaker et al. 1995; Zhang et al. 1997; Hoffland et al. 1989).

Citrate transporters that are known to transport free citrate as a chelating agent have been extensively studied in plants (Liu et al. 2009; Zhou et al. 2013; Yokosho et al. 2009; Yokosho et al. 2011). One particular family of citrate transporter that transports free citrate is the multidrug and toxic compound extrusion (MATE) protein family. Many of these family members, such as *OsFRDL1* in rice (Yokosho et al. 2009), *FRD3* in Arabidopsis (Durrett et al. 2007; Roschzttardtz et al. 2011), and *GmFRD3* and *GmFRD3b* in soybean (Rogers et al. 2009), function in the efficient translocation of iron by excreting citrate into the xylem. Other MATE family genes, such as *OsFRDL4* in rice (Yokosho et al. 2011), *AtMATE* in Arabidopsis (Liu et al. 2009), and *HvAACT1* in barley (Furukawa et al. 2007), play a role in secreting citrate into the rhizosphere to detoxify aluminums by chelating them.

Citrate transporters that recognize and/or transport metal-citrate complexes, such as iron-citrate complexes, have not been well studied in plants, whereas some have been identified in bacteria (Boorsma et al. 1996; Korithoski et al. 2005; Blancato et al. 2008; Lensbouer et al. 2008). One example is the citrate-metal hydrogen symport (CitMHS) family, which transports metal-citrate complexes in symport with a proton. This family of citrate transporters has evolved in some bacteria, such as *Bacillus*, *Streptomyces*, *Klebsiella*, and *Neisseria*. It is believed that these bacteria use metal–citrate complexes as a source of both carbon and metal, because metal–citrate complexes are predominantly accessible in their native environment, such as the rhizosphere (Dessureault-Rompré et al. 2008). It remains unknown if these CitMHS family transporters exist in eukaryotes.

Leaf variegation mutants have been reported in many species of higher plants (Li et al. 2010; Sakuraba et al. 2013; Martínez-Zapater 1993; Rédei 1967; Campitelli et al. 2008; Tsukaya et al. 2004). The leaves of these mutants have green and yellow (or white) sectors, and in most cases, this abnormal phenotype is caused by impaired chloroplast biogenesis in the off-colored cells (Aluru et al. 2006). While the green sectors of the leaves have morphologically normal plastids with the same levels of chlorophyll and carotenoids compared with wild-type leaves, the yellow (or white) sectors contain chloroplasts with defective thylakoid stacking and less photosynthetic pigments (Li et al. 2010; Han et al. 2012; Carol et al. 1999). In addition, leaf variegation can arise from excessive accumulation of reactive oxygen species (ROS), as is the case with *zebra-necrosis* (*zn*) mutants (Li et al. 2010) and *Staygreen* (*SGR*)-overexpressing plants in rice (Jiang et al. 2011), and *yellow variegated2* (*var2*) mutants in Arabidopsis (Kato et al. 2009).

Here, we investigated a new type of rice leaf variegation mutant, *zebra3* (*z3*), which has transverse dark-green/green stripes in the leaves with no yellow or white sectors. The dark green sectors of the *z3* mutant leaves had significantly higher levels of photosynthetic pigments and proteins compared with wild-type leaves, and the green sectors had levels similar to the wild type. Positional cloning revealed that the *Z3* gene encodes a protein predicted to be a CitMHS family citrate transporter, which have only been reported in bacteria thus far. Indeed, we found that the dark-green sectors accumulated more citrates than the green sectors, consistent with a putative role for Z3 in citrate transport. We discuss the possible function of Z3 as a candidate citrate transporter in eukaryotes.

## Results

### Phenotypic characterization of the *z3* mutant

**Fig. 1:**
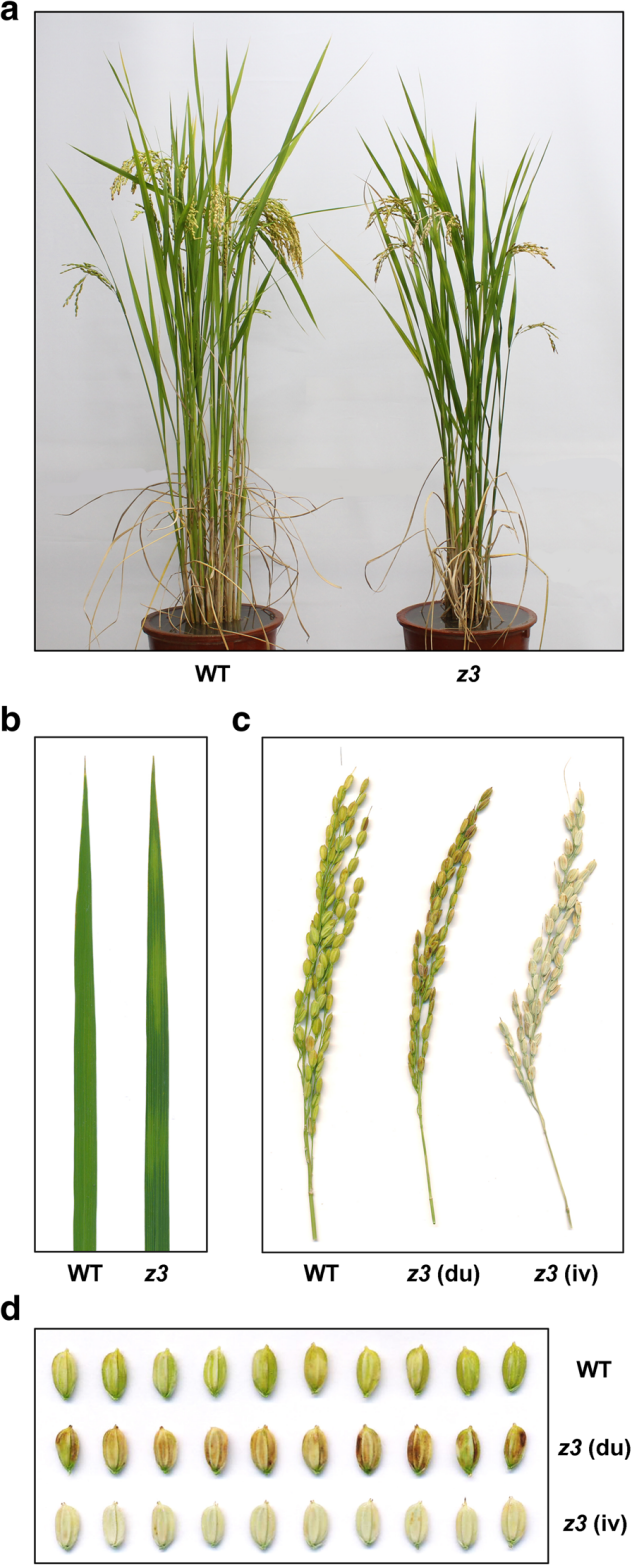
Phenotypic characterization of the *z3* mutant. **a** Phenotypes of the wild-type (WT) and *z3* mutant plants at 140 days after sowing (DAS) grown under natural long day conditions in the paddy field. **b** Leaf phenotypes of the 140-day-old WT and *z3* mutant plants grown in the paddy field. **c–d** Panicle (**c**) and grain (**d**) phenotypes of the WT and *z3* mutant plants at 140 DAS grown under natural long day conditions. WT, wild type; *z3*, *zebra3*; *z3* (du), dull-brown colored grain of the *z3* mutant; *z3* (iv), ivory colored grain of the *z3* mutant

The *z3* mutant produced slightly lighter green leaf blades and had a slower growth rate compared to its parental *japonica* cultivar ‘Kinmaze’ during early vegetative growth in a natural paddy. These phenotypes of the *z3* mutant disappeared gradually and no distinct differences in phenotype were observed at 80 days after sowing, although the height of the *z3* mutant was slightly shorter than that of the wild type, possibly due to its slower growth at the early stages (data not shown). Thereafter, transverse dark-green and green sectors began to appear in the mature leaves of the *z3* mutant (Fig. 1a and b). The leaf variegation became more obvious as the plants approached maturity and was most notable in the fully mature leaves during grain filling.

In addition to the leaf variegation, the *z3* mutant showed a late-flowering phenotype in the paddy field under natural long-day (NLD) conditions (~14 h light/day in Suwon, Korea, 37°N latitude) (Additional file 1: figure S1a-b). To investigate whether a reduction in vegetative growth rate or prolonged plastochron (i.e. the time between one leaf initiation and the next) causes the late-flowering phenotype in the *z3* mutant, we compared leaf emergence rates between the wild type and the *z3* mutant under long-day conditions (14.5 h-light, 30 °C/9.5 h-dark, 24 °C) in the growth chambers. The wild type flowered around 110 days after sowing (DAS), whereas the *z3* mutant flowered around 119 DAS. At heading, the total number of leaves on the main culm of the *z3* mutant was less than the wild type (Additional file 1: figure S1c). This observation demonstrates that the leaf emergence rate of the *z3* mutant is significantly lower than the wild type, indicating that the *z3* mutation retards the vegetative growth rate or prolongs the plastochron.

Moreover, the *z3* mutant simultaneously produced two distinct defective panicles on the same plant; one was a dull brown color and the other was an ivory color instead of the normal golden-yellow color in the wild type (Fig. 1c and d). Seed coat discoloration is an early indicator of poor quality seed, resulting in dull brown-colored grains (Bodalkar and Awadhiya 2014), which negatively affects grain yield, especially by decreasing the number of filled grains per panicle (or seed-setting rate) and 1000-grain weight (Phat et al. 2005). Furthermore, ivory-colored spikelets, which are generally caused by excessive transpiration during panicle growth, often result in low fertility in rice (Tsuda et al. 2000). To examine the relationship between the defective seed phenotypes and yield components in the *z3* mutant, we evaluated several agronomic traits under NLD conditions: main panicle length, number of panicles per plant, number of spikelets per main panicle, seed setting rate, 500-grain weight, and grain yield per plant (Additional file 2: figure S2). All of the examined traits were inferior in the *z3* mutant (Additional file 2: figure S2a-e), and the grain yield of the *z3* mutant was significantly lower compared with the wild type (Additional file 2: figure S2f). These results indicated that seed discoloration in the *z3* mutant negatively affects important yield traits in rice.

### The levels of photosynthetic pigments and proteins are altered in the leaves of the *z3* mutant

**Fig. 2:**
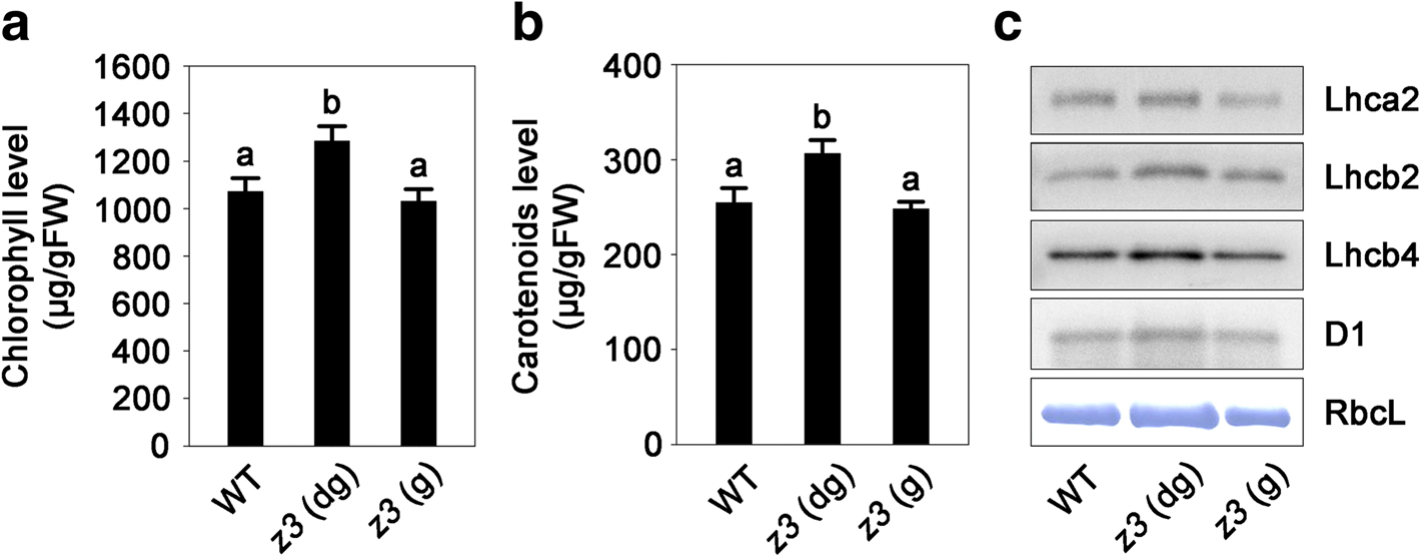
Characterization of photosynthetic pigments and proteins in the leaves of the *z3* mutant. **a–b** Chlorophyll concentrations (**a**) and carotenoids concentrations (**b**) in the leaves of the WT and the *z3* mutant. Chlorophyll and carotenoids were extracted from the second leaves of plants at 110 DAS grown under natural long day conditions. Means and SD were obtained from five biological replicates. The letters a and b on the bars denote that they are significantly different at the 1% level according to the Duncan’s multiple range test. **c** Immunoblot analysis of chloroplast proteins. Total protein was extracted from the second leaves of 110-day-old plants, and extracts were subjected to SDS-PAGE. Antibodies against Lhca2, Lhcb2, Lhcb4, and D1 were used for detection, and the Rubisco large subunit (RbcL) was detected by Coomassie brilliant blue staining. FW, fresh weight; WT, wild type; *z3* (dg), dark-green sectors of the *z3* mutant leaves; *z3* (g), green sectors of the *z3* mutant leaves

In typical leaf variegation mutants in rice, the relatively normal green sectors of the leaves are far lighter in color than the wild-type leaves. Consistent with the lighter color, typical leaf variegation mutants in rice also show substantial decreases in the concentrations of photosynthetic pigments and proteins, even in the green sectors of the leaves (Li et al. 2010; Han et al. 2012; Sakuraba et al. 2013). However, the relatively normal green colored sectors of the *z3* mutant were very similar in color to the wild-type leaves (Fig. 1b). Moreover, the levels of chlorophyll and carotenoids were higher than the wild type in the dark-green sectors of the *z3* mutant leaves, by approximately 19.6% and 20.4%, respectively, whereas their levels in the green sectors of the *z3* mutant leaves were statistically indistinguishable from the wild-type leaves (Fig. 2a and b). The levels of photosynthetic proteins (Lhca2, Lhcb2, Lhcb4, D1, and RbcL) showed similar patterns to those of the photosynthetic pigments (Fig. 2c) in that the quantities of all tested proteins were increased in the dark-green sectors of the *z3* mutant leaves, and the green sectors contained almost the same amount of photosynthetic proteins as the wild-type leaves (Fig. 2c). These results suggest that the *z3* mutation affects the levels of photosynthetic pigments and proteins in mature leaves in a transverse pattern.

### Map-based cloning of the *z3* locus

**Fig. 3:**
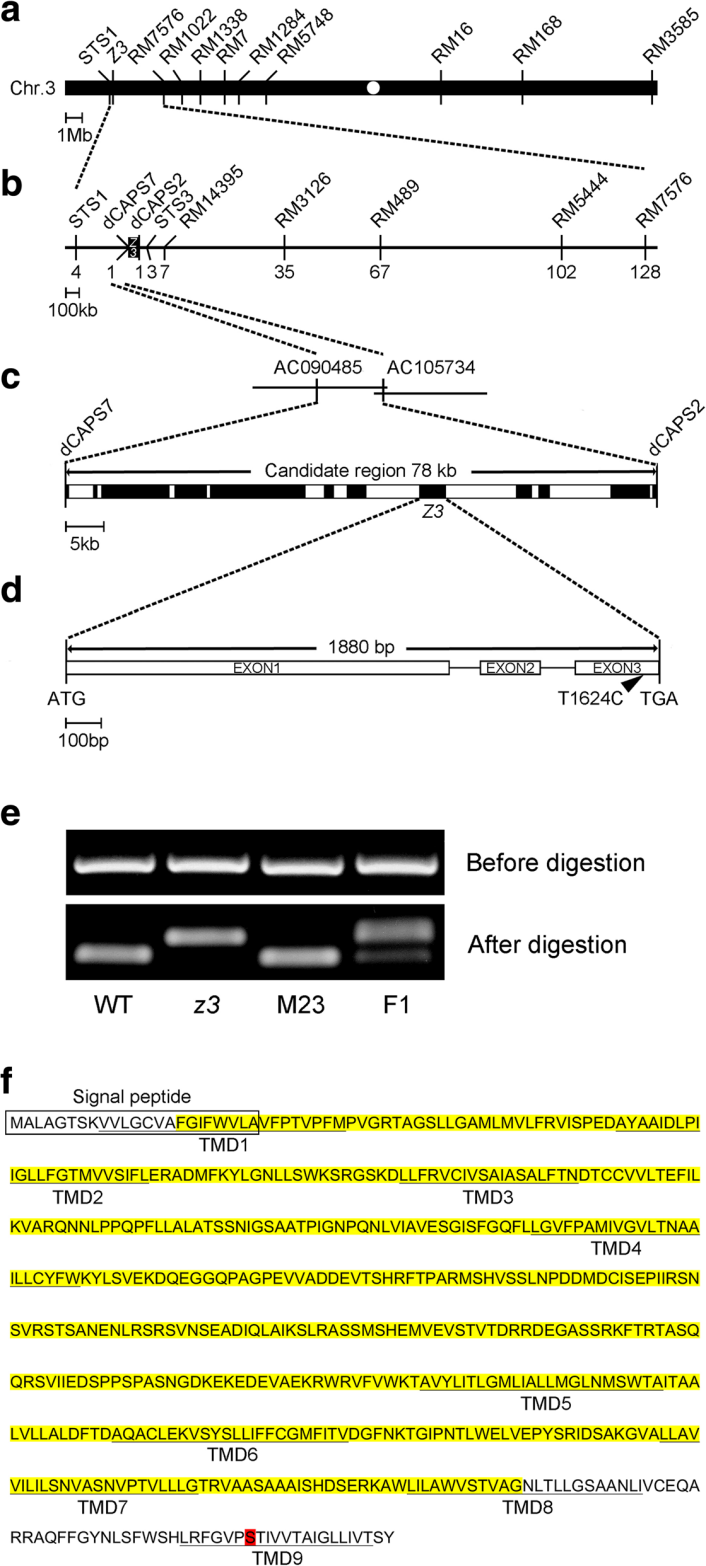
Map-based cloning of the *z3* locus. **a** Physical mapping of the *z3* locus. The *z3* locus was initially mapped to a 3.91-Mb region between two markers, STS1 and RM7576, on the short arm of chromosome 3. The marker information is listed in Additional file 8: Table S1. **b** Fine mapping of the *z3* locus. The locus was further mapped within a 78-kb region between two markers, dCAPS7 and dCAPS2. Numbers below the line indicate F_2_ recombinants at the marker regions. **c** Candidate genes (black boxes) in the 78-kb region. **d** T to C substitution of *Z3* in the *z3* mutant. Three exons and two introns are indicated as rectangles and lines, respectively. The position of the point mutation in the *z3* mutant is at the third exon and represented by a black arrowhead. **e** Derived cleaved amplified polymorphic sequence (dCAPS) analysis of the point mutation in the *z3* mutant. *Aat*ll was able to digest the genomic PCR products amplified from the WT, but not from the *z3* mutant because of its T to C substitution in the *Aat*ll restriction enzyme site. M23, a mapping parent ‘Milyang23’; F1, F_1_ hybrid (*z3*/Milyang23). **f** Z3 protein sequence. The predicted N-terminal signal peptide is marked with an open rectangle and the nine transmembrane domains (TMDs) are underlined. The yellow highlight represents regions predicted to be a CitMHS family. The position of the point mutation in the *z3* mutant is highlighted in red

The single recessive *z3* mutation was previously mapped onto the classical genetic map of the short arm of chromosome 3 (Iwata et al. 1979). To identify the *Z3* gene, we generated a mapping population of 1547 F_2_ plants from a cross between the *z3* mutant (*japonica*) and Milyang23 (an *indica/japonica* hybrid). A total of 291 plants among the F_2_ progenies clearly demonstrated the *z3* phenotype in the mature leaves and were used as the homozygous *z3* mutant genotype for map-based cloning. The *z3* locus was initially mapped to a 3.91-Mb region on the short arm of chromosome 3 using one sequence-tagged site (STS) and nine simple sequence repeat (SSR) markers (Fig. 3a). By fine mapping, the *z3* locus was further mapped to a 78-kb interval (Fig. 3b). Twelve predicted genes were identified in this region from the Rice Functional Genomic Express (RiceGE) database (Fig. 3c). To identify the *Z3* gene, we compared the sequence of the 78-kb candidate genomic region between the wild-type *japonica* cultivar ‘Nipponbare’ (NB) and the *z3* mutant. Of the twelve candidate genes, eleven open reading frames (ORFs) were exactly the same between NB and the *z3* mutant, and only one expressed a gene of unknown function (LOC_Os03g05390). This candidate gene contained a single base substitution from T to C in the third exon, leading to a missense mutation (serine to proline) at the 542th amino acid (Fig. 3d). This single-base substitution was further confirmed by dCAPS analysis (Fig. 3e).

To investigate the importance of a serine at the 542th amino acid (S542) for its role in protein function, we obtained a few protein sequences of predicted orthologs from higher plants using the NCBI-BLASTP program (https://blast.ncbi.nlm.nih.gov/Blast.cgi?PROGRAM=blastp&PAGE_TYPE=BlastSearch&LINK_LOC=blasthome) for amino acid alignment (Additional file 3: figure S3). This analysis revealed that the serine^542^ (S542) residue was highly conserved in higher plants including *Arabidopsis thaliana*, *Zea mays*, *Hordeum vulgare*, *Glycine max*, *Sorghum bicolor*, *Solanum tuberosum*, and *Oryza brachyantha*. This suggested that S542 is a critical residue in terms of the structure or function of the Z3 protein.

The *Z3* gene consists of a 1674-bp open reading frame encoding 557 amino acids with a molecular weight of 60.25 kDa. Z3 has a predicted N-terminal secretory pathway signal peptide, based on analysis with TargetP (Emanuelsson et al. 2000) (Fig. 3f, open rectangle). In addition, Z3 contains nine transmembrane domains (TMDs) and is predicted to be a CitMHS family citrate transporter by the Rice Genome Annotation Project database (http:// rice.plantbiology.msu.edu/), as previously described (Kawahara et al. 2013) (Fig. 3f). These data suggest that Z3 is an integral membrane protein.

**Fig. 4:**
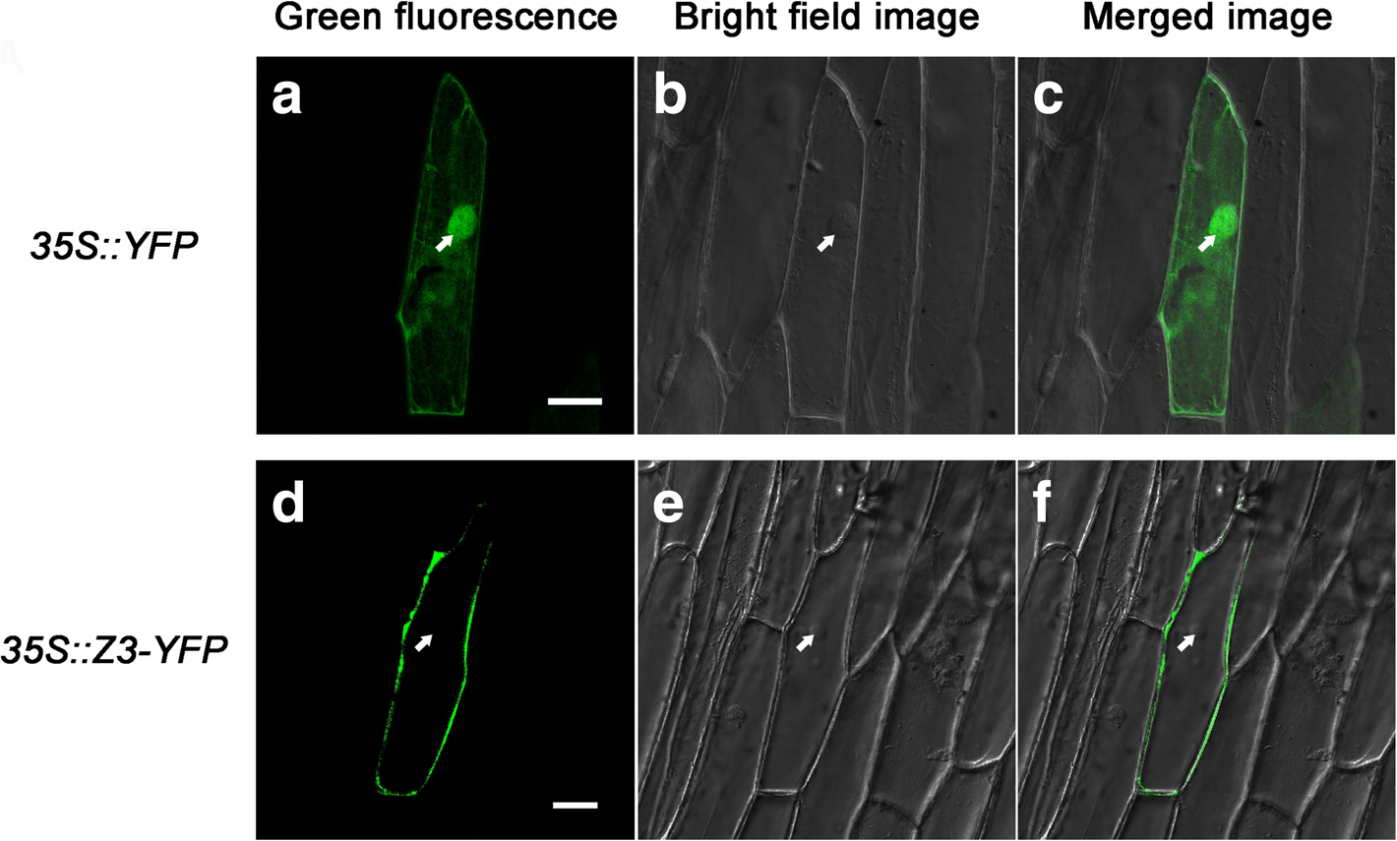
Z3 localizes in the plasma membrane. **a–c** Onion epidermal cells transformed with the control construct (*35S::YFP*). **d–f** Onion epidermal cells transformed with the construct *35S::Z3-YFP*. **a, d** Green fluorescence; **b, e** bright field image; **c, f** merged image. Cells were analyzed by confocal microscopy 18 h after the particle bombardment. Arrows indicate the nucleus. Scale bars = 50 μm

To determine the intracellular localization of Z3, a reporter gene encoding yellow fluorescent protein (YFP) was fused to the C-terminus of Z3, and the construct was introduced into onion epidermal cells using particle bombardment. The fluorescent signal of YFP alone, used as a control, was observed throughout the cells (Fig. 4a–c), whereas the signal of the Z3-YFP fusion protein was observed only at the plasma membrane (Fig. 4d–f). These results suggest that Z3 localizes in the plasma membrane.

### Complementation of the *z3* mutation by constitutive expression of *Z3* in Rice

**Fig. 5:**
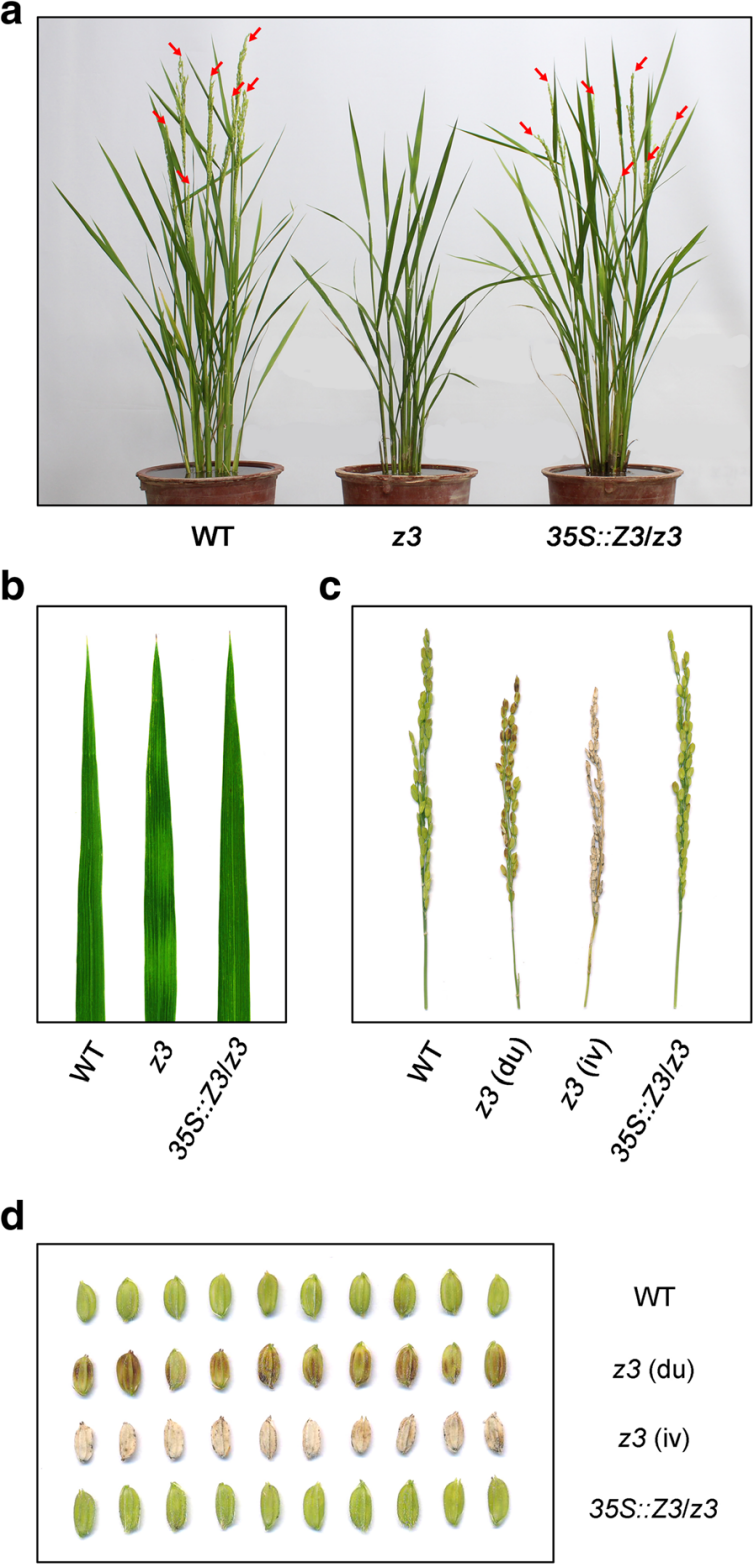
Complementation of the *z3* mutation. **a** Phenotypes of the WT, the *z3* mutant, and the *35S::Z3*/*z3* transgenic plant at 80 DAS grown under natural long day conditions. Leaf variegation and delayed flowering (Additional file 1: figure S1) of the *z3* mutation were rescued by the transformation of *35S::Z3* construct. WT flowered around one month earlier than the days to heading data in Additional file 1: figure S1 because the seeds of the WT, the *z3* mutant, and the *35S::Z3*/*z3* transgenic plant in Fig. 5a were sown on June, not in April (Suwon, Korea, 37°N latitude). Arrows indicate the panicle. **b** Leaf phenotypes of the WT, the *z3* mutant, and the *35S::Z3*/*z3* transgenic plant at 80 DAS grown in the paddy field. **c–d** Panicle and grain phenotypes of 80-day-old WT, *z3* mutant, and *35S::Z3*/*z3* transgenic plants grown under natural long day conditions. *z3* (du), dull-brown colored grain of the *z3* mutant; *z3* (iv), ivory colored grain of the *z3* mutant

To confirm that the missense mutation in Z3 (S542) is responsible for pleiotropic phenotype of the *z3* mutant, including leaf variegation, seed discoloration, and late flowering, we transformed the *z3* mutants with the full-length cDNA of *Z3* driven by the cauliflower mosaic virus 35S promoter (*35S::Z3*). We obtained six independent T_0_ transgenic plants and observed the phenotypes of T_1_ transgenic lines. To confirm the rice transformation, we performed PCR analysis using primers designed to amplify the *Z3* transgene and confirmed all six independent T_1_ transgenic plants (Additional file 4: figure S4a). Overexpression of *Z3* by the CaMV 35S promoter in the transgenic lines was validated by semi-quantitative RT-PCR (Additional file 4: figure S4b). The six complementation lines (*35S:Z3*/*z3*) grew normally at the early vegetative stage uren contrast to the inferior growth of the *z3* mutant (data not shown), and flowered as early as the wild type (Fig. 5a), indicating that constitutive expression of *Z3* complemented the late-flowering phenotype of the *z3* mutants under NLD conditions in the field. Moreover, the leaf and grain phenotypes of these six complementation lines were indistinguishable from those of the wild-type plants (Fig. 5b–d), confirming that the leaf variegation and seed discoloration phenotypes of the *z3* mutant results from the missense mutation in Z3 (S542).

To further validate the effects of the z*3* mutation on the defective leaf and seed phenotypes, we obtained two independent T-DNA insertional alleles for the *z3* mutation from the Salk Institute Genomic Analysis Laboratory (http://signal.salk.edu/cgi-bin/RiceGE) (Jeon et al. 2000). We designated these two independent alleles as *z3–2* and *z3–3*, and the *z3* allele identified by map-based cloning in this study as *z3–1*. For the *z3–2* and *z3–3* alleles, the T-DNA was inserted in the first exon of *Z3*, but in different regions (Additional file 5: figure S5a). We performed genotyping to confirm the T-DNA insertion in the *Z3* gene, and obtained homozygous mutants for each allele (Additional file 5: figure S5f). The absence of the full-length transcript of *Z3* in these two T-DNA lines was validated by semi-quantitative RT-PCR (Additional file 5: figure S5 g). The two independent *z3–2* and *z3–3* mutants began to produce leaf variegation at about 95 days after sowing, and transverse variegation of the leaf blades gradually became clearer thereafter, as in the *z3–1* mutant (Additional file 5: figure S5b). Furthermore, they also flowered late (Additional file 5: figure S5e) and exhibited two types of panicles on the same plant; an ivory colored head and a panicle with dull brown-colored grain (Additional file 5: figure S5c-d). These observations further demonstrated that the loss-of-function *z3* mutation is responsible for the pleiotropic phenotypes including leaf variegation, late flowering, and seed discoloration.

### The *Z3* gene is mainly expressed in the leaf blade and panicle branches at the heading stage

**Fig. 6:**
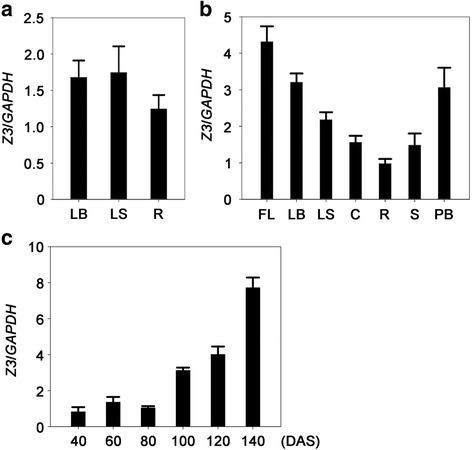
Spatial and temporal expression of the *Z3* gene. **a** Expression of the *Z3* gene in different organs at the seedling stage. Different organs were sampled from 2-week-old seedlings (cv Kinmaze) grown under long-day conditions (14.5 h-light/9.5 h-dark) in the growth chamber. LB, leaf blade; LS, leaf sheath; R, root. **b** Spatial expression of the *Z3* gene in different rice tissues at heading stage. Different tissues were sampled from the 115-day-old wild type grown under natural long-day conditions in the paddy field. FL, flag leaf; LB, leaf blade of the second leaf; LS, leaf sheath; C, culm; R, root; S, spikelet; PB, panicle branch. **c** Temporal expression of the *Z3* gene. Samples were harvested from fully expanded young leaves of the wild type at different stages grown under natural long -day conditions. DAS, days after sowing. *GAPDH* was used as internal control. All data in Fig. 6 are means ± SD (*n* = 3)

To examine the potential tissue specificity of Z3, we used qRT-PCR to characterize the spatial patterns of *Z3* expression at the seedling and heading stages. In the 2-week-old seedling plants, the *Z3* gene appeared to be expressed evenly in all tissues, including leaf blade, leaf sheath, and root (Fig. 6a). However, at the heading stage, *Z3* was mainly expressed in leaf blades and panicle branches, although we could detect *Z3* transcripts in all tissues, including flag leaf, second leaf blade, leaf sheath, culm, root, and spikelet (Fig. 6b).

Leaf variegation of the *z3* mutant appears around three months after sowing, approximately at the panicle differentiation stage, in natural long-day conditions. This suggests that the Z3 protein has a functional role in the mature leaf mainly at the late vegetative stage. To test whether *Z3* expression changes in the wild type when leaf variegation appears in the *z3* mutant, we inspected the transcript levels of the *Z3* gene in the leaf blade with qRT-PCR at different growth stages of the wild type grown in the field under natural long-day conditions at 20-day intervals. This showed that *Z3* expression in the wild-type leaves was upregulated at three months after sowing, and continued to increase thereafter (Fig. 6c).

### The leaf variegation in the *z3* mutant may be caused by different levels of citrate in the dark-green and green sectors

Excessive accumulation of ROS in leaves can lead to leaf variegation and/or necrosis in some rice mutants such as in the *preharvesting sprouting3* (*phs3*), *faded green leaf* (*fgl*), and *zebra2* (*z2*), *zebra-necrosis* (*zn*) mutants, and the *Staygreen* (*SGR*)-overexpressing plants (Han et al. 2012; Li et al. 2010; Sakuraba et al. 2013; Jiang et al. 2011; Fang et al. 2008). Therefore, we examined the levels of two types of ROS molecules, hydrogen peroxide and superoxide anion radicals, in the leaves of the *z3* mutant. Hydrogen peroxide and superoxide anion radicals were not detectable in the *z3* mutant leaves (Additional file 6: figure S6). These results suggested that ROS are not involved in the formation of variegated leaves in the *z3* mutant, unlike other leaf variegation mutants in rice.

Many leaf variegation mutants have defects in chloroplast development. For example, chloroplasts in the *fgl* mutant display defective thylakoid stacking and have several plastoglobules (Sakuraba et al. 2013), and chloroplasts in the *yellow-green leaf 2* (*ygl2*) and the naturally occurring Baihuaidio 7 mutants exhibit fewer lamellar structures (Chen et al. 2013; Li et al. 2012). To determine whether the leaves in the *z3* mutant show defects in chloroplast structure, we performed ultrastructural analysis of the chloroplasts in the leaves of the wild type and the *z3* mutant. The chloroplasts in the *z3* mutant leaves were indistinguishable from those of the wild type, with both exhibiting well-stacked grana thylakoids with no plastoglobules (Additional file 7: figure S7). Moreover, the green sectors of the *z3* mutant leaves contain almost the same levels of photosynthetic pigments compared with the wild type. These results indicated that the *z3* mutation has little effect on chloroplast development.

**Fig. 7:**
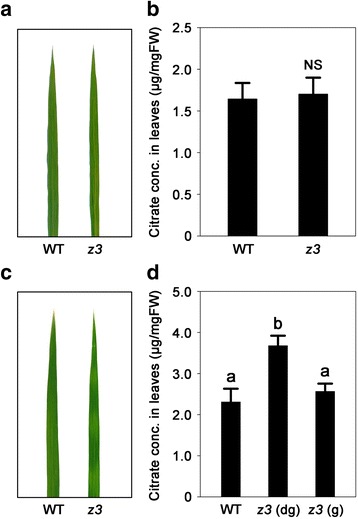
Citrate concentrations in the leaves of the *z3* mutant. **a, c** The second leaves of 90-day-old (**a**) and 117-day-old (**c**) WT and the *z3* mutant grown in a natural paddy field. **b, d** Citrate concentrations in the second leaves of the WT and the *z3* mutant at 90 DAS (**b**) and 117 DAS (**d**) grown under natural long day conditions. Citrate concentrations were determined with HPLC. Data are means ± SD (*n* = 4). Different letters indicate a significant difference at *P* < 0.01 (Duncan’s test). Abbreviations: WT, wild type; *z3* (dg), dark-green sectors of the *z3* mutant leaves; *z3* (g), green sectors of the *z3* mutant leaves; FW, fresh weight; NS, not significant; conc., concentration

In the NCBI BLAST (https://blast.ncbi.nlm.nih.gov/Blast.cgi) and the Rice Genome Annotation Project databases (http:// rice.plantbiology.msu.edu/), the Z3 protein is predicted to be a CitMHS family citrate transporter (Fig. 3f). This suggests that Z3 might be involved in the distribution and/or translocation of citrate in rice. Therefore, we measured citrate concentrations in the leaves of the wild type and the *z3* mutant using HPLC. The concentration of citrate in 90-day-old *z3* mutant leaves (prior to visible leaf variegation) was similar to that of the wild type (Fig. 7a–b). However, citrates accumulated to high levels in the dark-green sectors of 117-day-old *z3* mutant leaves, but not in the green sectors (Fig. 7c–d). Therefore, the citrate concentration in the leaves was not affected by the *z3* mutation before leaf variegation was visible. These results suggest that Z3 might play a role in the distribution and/or translocation of citrate in leaves after the late vegetative stage, and thus the *z3* mutant produces variegated leaves after 95 days after sowing, possibly by accumulating citrate to different extents in the dark-green and green parts of the leaves.

## Discussion

Here, we report a new type of leaf variegation mutant, *zebra3* (*z3*), which has transverse dark-green/green sectors in mature leaves without yellow or white sectors. In most cases, the leaves of variegated mutants have green/yellow (or white) sectors, and this abnormal phenotype is caused by impaired chloroplast biogenesis in the yellow or white sectors. Thus, the yellow (or white) sectors of these mutant’s leaves often contain chloroplasts with defective thylakoid stacking, less photosynthetic pigments, and also accumulate excessive levels of ROS (Li et al. 2010; Han et al. 2012; Carol et al. 1999). However, the *z3* mutant leaves did not show any of these defects. Even the slightly lighter green colored sectors in the *z3* mutant leaves contained similar levels of photosynthetic pigments and proteins to the wild type (Fig. 2), and showed normal chloroplast structure with well-stacked grana thylakoids (Additional file 7: figure S7) when compared with the wild type. Moreover, the *z3* mutant leaves did not accumulate excessive ROS as is common in other leaf variegation mutants (Additional file 6: Figure S6). These results suggest that leaf variegation of the *z3* mutant is not associated with a defect in chloroplast biogenesis or an increase of ROS.

The *z3* mutant is further different from other *zebra* mutants in that leaf variegation occurs at the late vegetative stage, around the panicle differentiation stage, strongly suggesting that Z3 has an important role after the late vegetative stage. Consistent with this, *Z3* expression increased in mature leaves at this stage, and its transcript level increased further thereafter (Fig. 6c). These results may partially explain why leaf variegation of the *z3* mutants is visible only at the late growth stages.

In addition, the *z3* mutant produced pale-green leaves (data not shown) and had a slower growth rate at the seedling stage (Additional file 1: figure S1c), although the overall phenotype of the *z3* mutant is normal compared with the wild type during vegetative growth. Even though Z3 mainly functions after the late vegetative stage, it also has a basal function in the early vegetative stages, since *Z3* is expressed in all tested tissues, including leaf blade, leaf sheath, and root (Fig. 6a). Taken together, these results indicate that Z3 protein may be involved in the maintenance of chlorophyll biosynthesis and overall growth rate at the early vegetative stage.

Map-based cloning revealed that *Z3* encodes a putative citrate transporter that belongs to the citrate-metal hydrogen symport (CitMHS) family. CitMHS family members transport metal–citrate complexes in symport with a proton, and have only been identified in bacteria thus far. Dessureault-Rompré et al. (2008) reported that bacteria acquire citrate and metal ions by importing metal-citrate complexes against the cellular electrochemical potential gradient by coupling import to the transport of a proton. In this scenario, citrate functions not only as an energy source but also as a chelating agent. In plants, citrate is mostly involved in cellular metabolism, such as the Krebs cycle, fatty acid synthesis, NH_4_^+^ assimilation, and the glyoxylate cycle (Sarantinopoulos et al. 2003; Krebs and Johnson 1937; Fritsch and Beevers 1979; Mattoo and Modi 1970; Nelson and Rinne 1975; Chen and Gadal 1990; Sienkiewicz-Porzucek et al. 2008; Tanner and Beevers 1965). Because the Z3 protein localizes to the plasma membrane (Fig. 4) and is predicted to be a CitMHS family member (Fig. 3), it may transport citrate among leaf cells. Furthermore, as the leaf blades of the *z3* mutant accumulated citrate to different extents in the dark-green and green sectors (Fig. 7), it appears that Z3 can act as a citrate transporter between leaf cells, and thus may play an important role in the balanced distribution of citrates. However, when citrate is released and transported outside the cell, it can function as a chelator. Therefore, the citrates potentially transported by Z3 may act as chelators, rather than as intermediates, in cellular metabolism. In this respect, it can be speculated that the unusual leaf variegation phenotype in the *z3* mutant is associated with metal ions which play a key role in chlorophyll biosynthesis. However, we cannot rule out the possibility that the leaf variegation in the *z3* mutants is caused by the citrates by themselves.

Leaf variegation mutants can be classified into two types depending on the genotypes of the green sectors and yellow (or white) sectors. First, in some mutants, the green and the yellow (or white) sectors in the leaves have different genotypes. That is, the green sectors contain the wild-type alleles, while the yellow (or white) sectors contain only mutant alleles. In many cases, this type of leaf variegation results from transposable element activity. For example, *dcl* (*defective chloroplasts and leaves*) in tomato (Keddie et al. 1996), *dag* (*differentiation and greening*) in *Antirrhinum* (Chatterjee et al. 1996), and *pyl-v* (*pale-yellow-leaf variegated*) in rice (Tsugane et al. 2006) are well-studied mutable alleles of genes for chloroplast biogenesis. In the second type of leaf variegation, all cells in the mutant leaves have the same genotype. That is, both green and yellow (or white) sectors contain the same mutant alleles, but the phenotype is differentially expressed between subsets of the cells, leading to the generation of green and yellow/white sectors. Because the leaves of the *z3* mutant have a uniform mutant genotype (data not shown), the leaf variegation of the *z3* mutant belongs to the second type. However, the leaf variegation mechanism of this type remains poorly understood.

One outstanding question about the *z3* phenotype is how mutation of a putative citrate transporter produces variegated leaves, although the all cells in the *z3* mutant have the same mutant genotype. The *z3* mutant leaves accumulated different levels of citrates in the dark-green and the green sectors (Fig. 7c-d), and this might be associated with the production of variegated leaves. As the Z3 protein is thought to transport citrates and the *z3* mutant accumulates the citrates to high levels in the dark-green sectors of leaves, it is plausible that the Z3 is involved in dispersion of citrates in the leaves. In this scenario, there might be other citrate transporters which are involved in polar transport of citrates in leaves. Polar transport is a directional transport of functional molecules between cells, and plasma membrane transporters that localize in a polar manner are responsible for polar transport in most cases (Naramoto 2017). For example, AtPIN1, an auxin efflux carrier protein in *Arabidopsis*, localizes only at one side of the plasma membrane in cells, and transports auxin to convergence points in the epidermal tissues where the major veins are produced (Scarpella et al. 2006). Similarly, there might be other citrate transporters, possibly such as MATE family citrate transporters (Yokosho et al. 2009; Yokosho et al. 2011) or other CitMHS family citrate transporters, which transport citrates to convergence points in leaves in transverse patterns after the late vegetative stage. In this case, citrate transported transversely by these citrate transporters could be dispersed by Z3 to maintain a uniform distribution of citrate in the leaves of the wild type. Therefore, it could be speculated that due to the functional deficiency of Z3 protein, the uniform citrate distribution in leaves is disrupted, resulting in accumulation of citrate in transverse patterns. The formation of dark green sectors and different citrate concentrations in the sectors of *z3* mutant leaves remains a key question for future study.

The *z3* mutant exhibits a pleiotropic phenotype in addition to leaf variegation; the *z3* mutants flowered late compared with the wild type (Additional file 1: figure S1a-b). *Z3* was expressed throughout the tested tissues, even in early vegetative stages (Fig. 6a) and *Z3* expression occurs throughout development (Fig. 6c), indicating that the Z3 protein has basal roles in all tissues. As the Z3 protein may transport metal-citrate complexes, we speculate that the *z3* mutation might disrupt metal ion homeostasis, thus affecting overall growth rate. Consistent with our speculation, the *z3* mutant produced slightly lighter green leaves at the seedling stage (data not shown), and showed inferior growth rates during vegetative growth in comparison with the wild type (Additional file 1: figure S1c). These results suggest that the late flowering of the *z3* mutant is partially due to the retardation of growth resulting from unbalanced distribution of metal ions. However, based on our current data, we cannot rule out the possibility that the *z3* mutation also causes a delayed floral transition.

The *z3* mutant also showed a grain discoloration phenotype at the ripening stage under natural long-day conditions (Fig. 1c-d). Grain discoloration in rice is characterized by dull brown or black spots on the husk, and reduces grain quality (Navasero and Winslow 1987). In most cases, fungal or bacterial infections are responsible for grain discoloration (Ngala 1983), and deficiency of mineral nutrients, such as silicon, is involved in this symptom (Datnoff et al. 1991; Winslow 1992). In addition, Dobermann and Fairhurst (2000) reported that the lack of metal ions, such as potassium, could cause irregular brown necrotic spots on developing panicles in rice. As *Z3* is highly expressed in panicle branches, similar to the expression levels in leaf blade (Fig. 6b), it is plausible that the Z3 protein also functions in translocating metal ions into grains. In this case, a functional deficiency of Z3 protein may disrupt the translocation of metal ions complexed with citrates into developing grains, and the resulting deficiency of metal ions, or possibly citrate itself, causes grain discoloration.

## Conclusions

The *zebra* mutants in rice produce transverse sectors of green/yellow or green/white in developing or mature leaves. In most cases, this abnormal phenotype is caused by impaired chloroplast biogenesis or excessive accumulation of ROS in the yellow or white sectors. In contrast to these mutants, the *zebra3* (*z3*) mutant has transverse dark-green/green stripes in the leaves with no yellow or white sectors. In addition, the *z3* mutant flowers about 10 days later than the WT, and produces defective panicles. We found that he *Z3* locus encodes a putative citrate transporter by map-based cloning and complementation test. Though the *z3* mutation has little effect on chloroplast development, citrate levels were significantly higher in the dark-green sectors of the *z3* mutant leaves than in the green sectors or the normal leaves of the wild type. These results indicate that leaf variegation in the *z3* mutant is caused by an unbalanced distribution of citrate in a transverse pattern in leaf tissues. Taking all the results together, we propose that Z3 plays a role in the transport and distribution of citrates in leaves, and is a possible candidate for CitMHS family members in higher plants.

## Methods

### Plant materials and growth conditions

The *zebra3–1* (*z3–1*) mutant was isolated from a mutant pool produced by applying *N*-methyl-*N*-nitrosourea, to a *japonica* rice cultivar ‘Kinmaze’, as previously described (Iwata et al. 1979). T-DNA knockout mutants of *Z3* (LOC_Os03g05390; PFG_1E-02030.R, designated as *z3–2*; PFG_2D-00576.R, designated as *z3–3*) were derived from the Korean *japonica* rice cultivar ‘Hwayoung’ (HY) and obtained from the Salk Institute Genomic Analysis Laboratory (http://signal.salk.edu/cgi-bin/RiceGE) (Jeon et al. 2000). All rice plants were grown under natural long day conditions (approximately 14 h light/day) in a paddy field (Suwon, Korea, 37°N latitude). For spatial expression analysis of the *Z3* gene at the seedling stage, the rice cultivar Kinmaze was grown under long-day conditions (14.5 h-light, 30 °C/9.5 h-dark, 24 °C) under cool-white light (300 μmol m^−2^ s^−1^) in the growth chamber.

### Leaf emergence rate and heading date

To compare the leaf emergence rate between the *z3* mutant and the wild-type, the plants were grown in long-day conditions (14.5 h-light, 30 °C/9.5 h-dark, 24 °C) with 60% relative humidity. The light source was light-emitting diodes producing mixed red, green, and blue light, and photon flux density was about 300 μmol m^−2^ s^−1^. Leaf emergence rate was estimated as previously described (Itoh et al. 1998). Briefly, when each new leaf blade had completely emerged from the sheath of the previous leaf, the number of leaves on the main culm was counted for each plant. Heading date (or flowering time) was recorded from sowing to emergence of the first panicle in the main culm.

### Map-based cloning

A mapping population of 1547 F_2_ individuals was generated by crossing a *japonica*-type *z3* mutant and a tongil-type cultivar, Milyang23 (M23). To confirm the chromosomal localization of the *Z3* locus, we initially mapped the *z3* mutation using 291 F_2_ plants with the *z3* mutant phenotype, 9 simple sequence repeat (SSR) markers, and one sequence-tagged site (STS) marker distributed on chromosome 3. SSR marker information is available in GRAMENE (http://www.gramene.org). For fine mapping, one STS and two derived cleaved amplified polymorphic sequence (dCAPS) markers were designed by comparison of the genomic DNA sequences between M23 and the *z3* mutant using whole-genome re-sequencing data (Additional file 8: Table S1). Genomic DNA was extracted from the leaf blades of two-week-old M23 and *z3* mutant plants using the NucleoSpin Plant II kit (Macherey-Nagel, Germany) according to the manufacturer’s instructions. Whole-genome re-sequencing was carried out using SolexaQA package v.1.13 (Cox et al. 2010) and BWA 0.6.1-r104 (Li and Durbin 2009). For dCAPS analysis, a mismatch forward primer (5′-CCACCTCCGGTTCGG GGTGAC-3′) and the reverse primer (5′- CAAAGGGTTCGTTCATCTATCTC -3′) were designed using DCAPS FINDER2.0 (http://helix.wustl.edu/dcaps/dcaps.html). Each PCR product was digested with a restriction enzyme, *Aat*II, and separated on an agarose gel.

### Subcellular localization of Z3

*YFP* cDNA was fused to the C-terminus of *Z3* cDNA in the pEarleyGate 101 (pEG101) vector through LR recombination (Lambda integrase/excisionase; Elpis-Biotech), resulting in the *35S:Z3-YFP* plasmid. The fusion construct, as well as the control (empty pEG101 vector; *35S:YFP*), were transformed into onion (*Allium cepa*) epidermal cells using a DNA particle delivery system (Biolistic PDS-1000/He; Bio-Rad, Hercules, CA, USA). The transformed onion epidermal layers were incubated at 25 °C on Murashige and Skoog medium (pH 5.7) plates under the dark for 20 h. Then, the onion cell layers were examined with a confocal laser scanning microscope (SP8 X, Leica, Germany).

### Vector construction and plant transformation

For complementation of the *z3* mutation, a full-length cDNA of *Z3* was cloned into the pMDC32 Gateway binary vector containing the cauliflower mosaic virus (CaMV) 35S promoter (Curtis and Grossniklaus 2003). The *35S:Z3* construct in the pMDC32 vector was introduced into calli generated from mature seed embryos of *z3* mutants through the *Agrobacterium* (strain EHA105)-mediated method (Jeon et al. 1999; Lee et al. 1999). The transgenic rice plants were selected on 2 N6 media containing hygromycin (50 mg L^−1^) and confirmed by PCR using the specific primer set, *Z3* (Transgene-2) F/R (Additional file 8: Table S1).

### Identification of the T-DNA insertional alleles *z3–2* and *z3–3*

To identify the homozygous *z3–2* and *z3–3* mutants, we extracted genomic DNA from the segregating T_1_ populations using a cetyl trimethyl ammonium bromide (CTAB) method (Murray and Thompson 1980) and performed PCR analysis. PCR was conducted with a T-DNA plasmid pGA2707 right border primer (*z3–2* RB) in combination with a *Z3* left genomic primer (*z3–2* LP) for the *z3–2* mutant, and with a T-DNA plasmid pGA2772 right border primer (*z3–3* RB) in combination with a *Z3* left genomic primer (*z3–3* LP) for the *z3–3* mutant (Additional file 8: Table S1). PCR was performed with 30 cycles of 98 °C for 30 s, 55 °C for 30 s, and 72 °C for 45 s. PCR was also conducted with a *Z3* left genomic primer (*z3–2* LP) in combination with a *Z3* right genomic primer (*z3–2* RP) for the *z3–2* mutant, and with a *Z3* left genomic primer (*z3–3* LP) in combination with a *Z3* right genomic primer (*z3–3* RP) for the *z3–3* mutant (Additional file 8: Table S1). PCR was performed with 30 cycles of 98 °C for 30 s, 55 °C for 30 s, and 72 °C for 1 min.

### Reverse transcription and semi-quantitative and quantitative RT-PCR

Total RNA was extracted from leaves using the MG Total RNA Extraction Kit (Macrogen, Seoul, Korea) according to the manufacturer’s instructions. First-strand cDNAs were synthesized from 2 μg of total RNA using oligo(dT)_15_ primers and M-MLV reverse transcriptase (Promega, Madison, WI, USA) and diluted with water to 100 μL. The transcript level of the *Z3* gene was examined by semi-quantitative RT-PCR using specific primers, and *Ubiquitin5* (*UBQ5*) (GenBank accession number: AK061988) or *Glyceraldehyde-3-phosphate dehydrogenase* (*GAPDH*) (GenBank accession number: AK064960) was used for a loading control as previously described (Jain et al. 2006).

To determine the transcript levels of the *Z3* gene by quantitative RT-PCR analysis, 20 μl of qRT-PCR mixture containing 2 μl of the first-strand cDNA mixture, 0.25 μM of the forward and reverse primers for each gene, and 10 μl of 2X QuantiTect LightCycler 480 SYBR Green I Master (Roche) were used. qRT-PCR analysis was performed on the Light Cycler 2.0 instrument (Roche Diagnostics, Germany). The mRNA levels of the *Z3* gene were normalized to those of *Glyceraldehyde-3-phosphate dehydrogenase* (*GAPDH*). Primer information is listed in Additional file 8: Table S1.

### Measurement of photosynthetic pigments

To evaluate the concentration of total chlorophyll and carotenoids, pigments were extracted from equal fresh weights of leaves with 80% ice-cold acetone. Chlorophyll and carotenoid levels were measured with a UV/VIS spectrophotometer (BioTek) as previously described (Lichtenthaler 1987).

### SDS-PAGE and immunoblot analysis

Photosynthesis-related proteins were detected as previously described, with some modifications (Han et al. 2012; Kwon et al. 2015; Kwon et al. 2017). Leaf tissue (10 mg) was homogenized with 100 μL of SDS-PAGE sample buffer [50 mM Tris pH 6.8, 2 mM EDTA, 10% (*w*/*v*) glycerol, 2% SDS, and 6% 2-mercaptoethanol] and denatured at 100 °C for 5 min, then samples were subjected to SDS-PAGE. For immunoblot analysis, 5 μL of each protein sample was used. The resolved proteins were electroblotted onto the Immobilon-P Transfer Membrane (Millipore). Antibodies against photosystem proteins (Lhca2, Lhcb2, Lhcb4, and D1) were obtained from Agrisera (Sweden). To detect the horseradish peroxidase activity of secondary antibodies (Sigma), an ECL detection kit, WESTSAVE (AbFRONTIER, Korea), and a chemiluminescence system (FUSION-FX7, France) were used according to the manufacturers’ protocols. The Rubisco large subunit (RbcL) was visualized by staining with Coomassie brilliant blue reagent (Sigma-Aldrich) after immunoblot analysis.

### Detection of reactive oxygen species

Detection of hydrogen peroxide (H_2_O_2_) and superoxide anion radicals (O_2_^−^) was carried out as previously described (Li et al. 2010; Han et al. 2012; Wi et al. 2010) with minor modifications. Hydrogen peroxide and superoxide anion radicals were detected by 3,3-diaminobenzidine (DAB) and nitroblue tetrazolium chloride (NBT), respectively. Flag leaves of 160-day-old plants grown in the field were sampled and incubated in 0.1% DAB (Sigma) in distilled water or 0.05% NBT (Duchefa) in 50 mM sodium phosphate buffer (pH 7.5) at room temperature overnight with gentle shaking. Chlorophyll was then removed by incubation in 90% ethanol at 80 °C. Hydrogen peroxide and superoxide anion radicals were visualized as reddish brown and dark blue stains, respectively.

### Transmission electron microscopy

Leaf samples for transmission electron microscopy were harvested from five-month-old plants grown under natural long day conditions. Fixation and polymerization of leaf samples were carried out as described previously (Park et al. 2007). Segments of leaf tissues were fixed in modified Karnovsky’s fixative (2% paraformaldehyde, 2% glutaraldehyde, and 50 mM sodium cacodylate buffer, pH 7.2) and washed three times with 50 mM sodium cacodylate buffer (pH 7.2) at 4 °C for 10 min. The samples were post-fixed with 1% osmium tetroxide in 50 mM sodium cacodylate buffer, pH 7.2, at 4 °C for 2 h and briefly washed twice with distilled water at 25 °C. The samples were then bloc stained in 0.5% uranyl acetate at 4 °C for a minimum of 30 min, dehydrated in a gradient series of ethanol and propylene oxide, and embedded in Spurr’s resin. After polymerization at 70 °C for 24 h, the sections were sliced to 60 nm with an ultramicrotome (MT-X; RMC, http://www.rmcboeckeler.com/) and stained with 2% uranyl acetate for 5 min and Reynold’s lead citrate for 2 min at 25 °C. The processed samples were finally examined using a JEM-1010 EX electron microscope (JEOL, https://www.jeol.co.jp/en/).

### Estimation of citrates by high performance liquid chromatography (HPLC)

Organic acids, including citrate, were extracted from leaf samples as previously described (Phillips and Jennings 1976), with some modifications. Leaf tissues (100 mg) of 117-day-old plants grown under NLD conditions were sampled and homogenized with 1 mL of cold acidified 80% ethanol. After centrifugation at 5000 g for 15 min, the supernatant was decanted and kept. All operations up to this point were carried out at 4 °C. The samples were filtered with a Millex-GV syringe filter (0.22 μm), and citrate concentrations in the samples were analyzed with HPLC using an Aminex 87H column. The mobile phase was a 0.01 N H_2_SO_4_ run at 40 °C, and peaks were detected by a refractive index detector (ERC, RefractoMAX520, Japan) at a wavelength of 210 nm.

## Additional files

Additional file 1: Figure S1. Late flowering phenotypes of the *z3* mutant. a Flowering phenotypes of the 117-day-old WT and *z3* mutant grown under natural long day conditions (14 h light/day, 37o N latitude) in the paddy field. b Days to heading of the WT and the *z3* mutant in natural long day conditions. Means and SD were obtained from 15 plants of each genotype. Error bars indicate SD. Differences between means were compared using Student’s *t*-test (*** *P* < 0.001). c Comparison of leaf emergence rates between the wild type and the *z3* mutants grown under long-day conditions (14.5 h-light/9.5 h-dark) in the growth chamber. Mean and standard deviation values are shown (*n* = 10). Leaf emergence rate was calculated according to the methods described by Itoh et al. (1998). The average heading dates of the wild type and the *z3* mutants are shown by closed and open arrows, respectively. (PDF 961 kb)

Additional file 2: Figure S2. Statistical analysis of agronomic traits in the *z3* mutant. a–f Agronomic traits were investigated between the WT and the *z3* mutant plants grown under natural conditions. Investigated traits are as follows: a main panicle length, b number of panicles per plant, c number of spikelets per main panicle, d seed setting rate, e 500-grain weight, and f yield per plant. Ten plants were used to measure each trait. Values are shown as means. Error bars indicate SD. Student’s *t*-test was performed for statistical analysis (* *P* < 0.05, ** *P* < 0.01, *** *P* < 0.001). (PDF 2110 kb)

Additional file 3: Figure S3. Amino acid alignment of Z3 homologs in higher plants. The amino acid sequences of Z3 homologs in higher plants were acquired from NCBI (http://www.ncbi.nlm.nih.gov/), and the amino acid alignment was obtained using the Clustal Omega EMBL-EBI (https://www.ebi.ac.uk/Tools/msa/clustalo/) and BoxShade 3.21 Server (https://www.ch.embnet.org/software/BOX_form.html). *O. brachyantha*, *Z. mays*_1, *Z. mays*_2, *H. vulgare*, *S. bicolor*, *S. tuberosum*, *G. max*, and *A. thaliana* have 99, 96, 95, 87, 72, 65, 64, and 62% sequence similarity to Z3, respectively. The mutated residue (S542P in *z3*) is indicated by a red arrowhead. *O. sativa*_*ZEBRA3* (*Oryza sativa* ZEBRA3, LOC_Os03g05390, NP_001048962.1); *O. brachyantha* (*Oryza brachyantha*, XP_006649400.1); *Z. mays*_1 (*Zea mays*, NP_001151517.1); *Z. mays*_2 (*Zea mays*, ACG43196.1); *H. vulgare* (*Hordeum vulgare*, BAK05230.1); *S. bicolor* (*Sorghum bicolor*, XP_002467148.1); *S. tuberosum* (*Solanum tuberosum*, XP_006363328.1); *G. max* (*Glycine max*, XP_003533988.1); *A. thaliana* (*Arabidopsis thaliana*, NP_171728.2). (PDF 2499 kb)

Additional file 4: Figure S4. Confirmation of the overexpression of *Z3* by the CaMV 35S promoter in six independent complementation lines. a Genomic PCR analysis for confirmation of rice transformation. The genomic region between the vector and the *Z3* transgene was amplified in the transformed lines. All six independent lines were confirmed to be transformed. Primer information is listed in Additional file 8: Table S1. EV, empty vector; Control, vector used for transformation without the *Z3* gene. b The overexpression of *Z3* by the CaMV 35S promoter in six independent complementation lines. Primers were designed to amplify the transgene mRNA, and are listed in Additional file 8: Table S1. The *GAPDH* mRNA level was measured as a loading control. (PDF 647 kb)

Additional file 5: Figure S5. Phenotypes of the homozygous T-DNA null mutants of *Z3*. a Schematic diagrams of the T-DNA insertion. The *z3–2* and *z3–3* mutants have a T-DNA insertion in the first exon, but in different locations. These T-DNA knockout mutants of *Z3* were derived from the *japonica* rice cv ‘Hwayoung’. Filled boxes and thick lines indicate exons and introns, respectively. Primers LP1 (left primer 1), RP1 (right primer 1), LP2 (left primer 2), RP2 (right primer 2), and RB (right border primer) used for genotyping are represented as arrows and listed in Additional file 8: Table S1. b Phenotypes of leaf blades from four-month-old WT (Hwayoung), and *z3–2* and *z3–3* mutants grown under natural long day (NLD) conditions in the paddy field. c–d Panicle and grain phenotypes of the WT (Hwayoung) and two *z3*-knockout mutants at 150 DAS grown in a paddy field. e Days to heading data of the WT, and the *z3–2*, and *z3–3* mutants in natural long-day conditions. Means and standard deviations were obtained from 5 plants of each genotype. Error bars indicate SD. Differences between means were compared using Student’s *t*-test (*** *P* < 0.001). f Genotyping of T-DNA insertion lines. Each LP and RP primer should have amplified about 1.1 kb of genomic DNA if no T-DNA insertion was made. If T-DNA was inserted, the length between the two primers was too large to be amplified. The RB and each RP primer should have amplified an approximately 0.6-kb band if T-DNA was inserted in the first exon. WT (Hwayoung) was used for the control. g Expression of *Z3* in the WT and T-DNA knockout mutants by semi-quantitative RT-PCR. *Z3* transcripts were not detected in the T-DNA knockout mutants. *Ubiquitin 5* (*UBQ5*) mRNA was measured as a loading control. db, dull-brown colored grain; iv, ivory colored grain. (PDF 1621 kb)

Additional file 6: Figure S6. Analysis of reactive oxygen species (ROS) in the *z3* mutant leaves. a-b Hydrogen peroxide (H2O2) and superoxide anion radicals (O2-) in flag leaves of the WT and *z3* mutants at 160 DAS grown under natural long day conditions were visualized by staining with DAB (a) and NBT (b), respectively. Leaves before (left) and after (right) staining are shown. (PDF 497 kb)

Additional file 7: Figure S7. Transmission electron microscopy analysis of chloroplasts in the *z3* mutant leaves. a-c Chloroplasts in the green leaves of the WT (a) and in the dark-green (b) and green (c) sectors of the *z3* mutant leaves. Flag leaves of the 150-day-old WT and *z3* mutant grown under natural long day conditions were sampled for analysis. G, grana thylakoid. Scale bars = 0.5 μm. (PDF 955 kb)

Additional file 8: Table S1. Primers used in this study. (PDF 1453 kb)

## Abbreviations

CaMV: Cauliflower mosaic virus
CitMHS: Citrate-metal hydrogen symport
CTAB: Cetyl trimethyl ammonium bromide
DAB: 3,3-Diaminobendizine
DAS: Days after sowing
dCAPS: Derived cleaved amplified polymorphic sequence
DTH: Days to heading
FW: Fresh weight
G: Grana thylakoid
GAPDH: Glyceraldehyde-3-phosphate dehydrogenase
GOGAT: Glutamate synthase
GS: Glutamine synthetase
HPLC: High performance liquid chromatography
HY: Hwayoung
KO: Knock-out
Lhca: Light-harvesting chlorophyll *a/b*-binding protein of photosystem I
Lhcb: Light-harvesting chlorophyll *a/b*-binding protein of photosystem II
LP: Left genomic primer
M23: Milyang23
MNU: *N*-methyl-*N*-nitrosourea
NB: Nipponbare
NBT: Nitroblue tetrazolium chloride
NLD: Natural long day
ORF: Open reading frame
PCR: Polymerase chain reaction
RB: Right border primer
ROS: Reactive oxygen species
RP: Right genomic primer
SD: Standard deviation
SDS-PAGE: Sodium dodecyl sulfate-polyacrylamide gel electrophoresis
SSR: Simple sequence repeat
STS: Sequence-tagged site
TEM: Transmission electron microscopy;
TMD: Transmembrane domain
*UBQ5*: *Ubiquitin5*
WT: Wild type
YFP: Yellow fluorescent protein
*Z3*: *ZEBRA3*
