# Supplementary material for: The rice zebra3 (z3) mutation disrupts citrate distribution and produces transverse dark-green/green variegation in mature leaves
Source: Rice (N Y). 2018 Jan 5;11:1. doi: 10.1186/s12284-017-0196-8 (PMC5756232; doi:10.1186/s12284-017-0196-8)
Supplement: Supplementary file 1 — Late flowering phenotypes of the z3 mutant. a Flowering phenotypes of the 117-day-old WT and z3 mutant grown under natural long day conditions (14 h light/day, 37o N latitude) in the paddy field. b Days to heading of the WT and the z3 mutant in natural long day conditions. Means and SD were obtained from 15 plants of each genotype. Error bars indicate SD. Differences between means were compared using Student’s t-test (*** P < 0.001). c Comparison of leaf emergence rates between the wild type and the z3 mutants grown under long-day conditions (14.5 h-light/9.5 h-dark) in the growth chamber. Mean and standard deviation values are shown (n = 10). Leaf emergence rate was calculated according to the methods described by Itoh et al. (1998). The average heading dates of the wild type and the z3 mutants are shown by closed and open arrows, respectively. (PDF 961 kb) [file 12284_2017_196_MOESM1_ESM.pdf]

Additional Files – Kim et al.

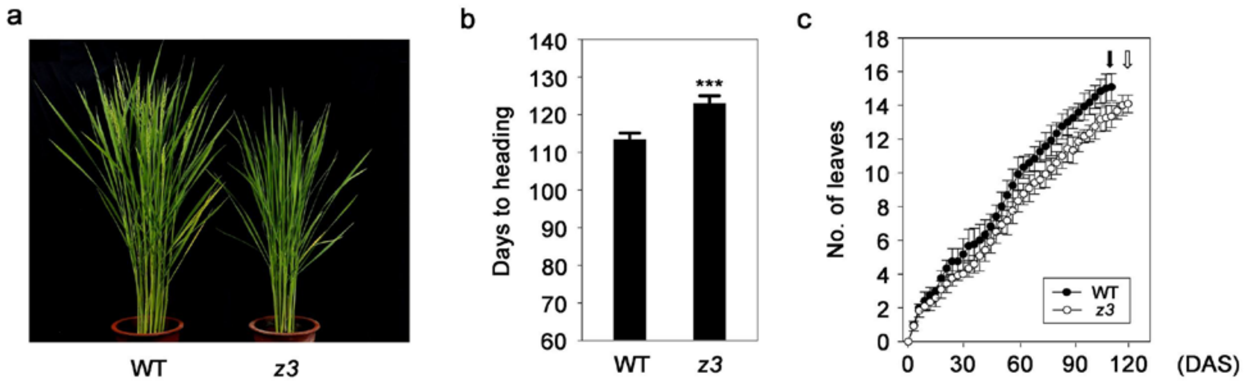

Additional File 1: Fig. S1 Late flowering phenotypes of the *z3* mutant.

a Flowering phenotypes of the 117-day-old WT and *z3* mutant grown under natural long day conditions (14 h light/day, 37° N latitude) in the paddy field. **b** Days to heading of the WT and the *z3* mutant in natural long day conditions. Means and SD were obtained from 15 plants of each genotype. Error bars indicate SD. Differences between means were compared using Student's *t*-test (*** $P < 0.001$). **c** Comparison of leaf emergence rates between the wild type and the *z3* mutants grown under long-day conditions (14.5 h-light/9.5 h-dark) in the growth chamber. Mean and standard deviation values are shown (n=10). Leaf emergence rate was calculated according to the methods described by Itoh et al. (1998). The average heading dates of the wild type and the *z3* mutants are shown by closed and open arrows, respectively.
