# Supplementary material for: The rice zebra3 (z3) mutation disrupts citrate distribution and produces transverse dark-green/green variegation in mature leaves
Source: Rice (N Y). 2018 Jan 5;11:1. doi: 10.1186/s12284-017-0196-8 (PMC5756232; doi:10.1186/s12284-017-0196-8)
Supplement: Supplementary file 2 — Statistical analysis of agronomic traits in the z3 mutant. a–f Agronomic traits were investigated between the WT and the z3 mutant plants grown under natural conditions. Investigated traits are as follows: a main panicle length, b number of panicles per plant, c number of spikelets per main panicle, d seed setting rate, e 500-grain weight, and f yield per plant. Ten plants were used to measure each trait. Values are shown as means. Error bars indicate SD. Student’s t-test was performed for statistical analysis (* P < 0.05, ** P < 0.01, *** P < 0.001). (PDF 2110 kb) [file 12284_2017_196_MOESM2_ESM.pdf]

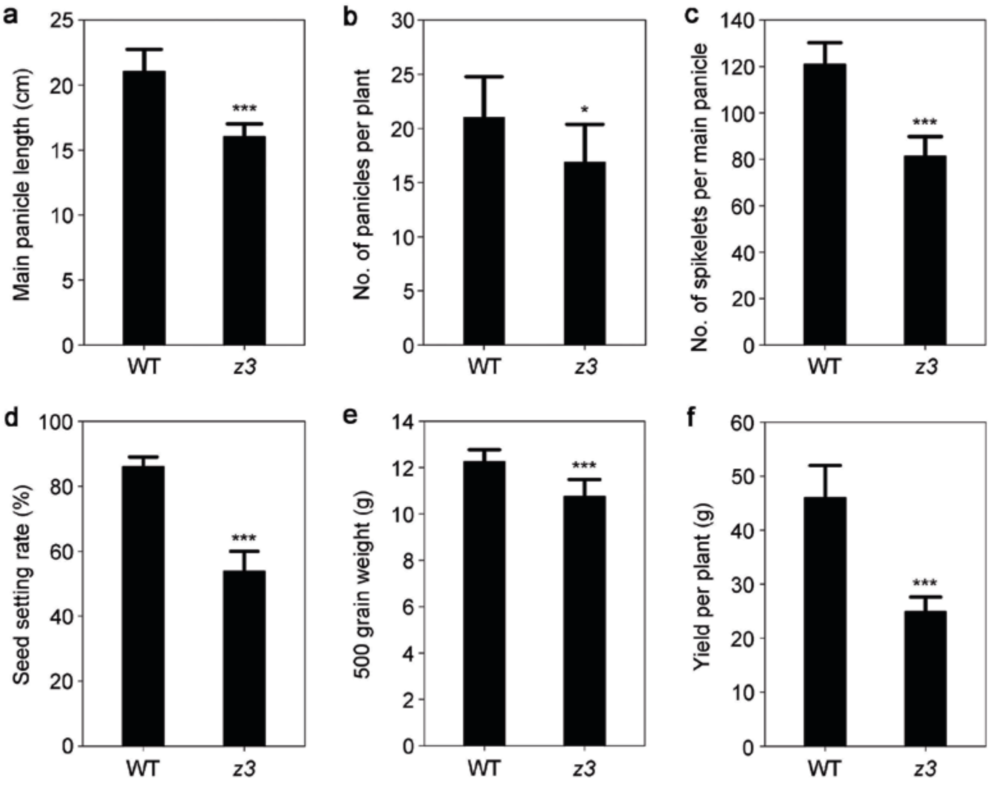

Additional File 2: Fig. S2 Statistical analysis of agronomic traits in the *z3* mutant.

a–f Agronomic traits were investigated between the WT and the *z3* mutant plants grown under natural conditions. Investigated traits are as follows: **a** main panicle length, **b** number of panicles per plant, **c** number of spikelets per main panicle, **d** seed setting rate, **e** 500-grain weight, and **f** yield per plant. Ten plants were used to measure each trait. Values are shown as means. Error bars indicate SD. Student's *t*-test was performed for statistical analysis (* $P < 0.05$, ** $P < 0.01$, *** $P < 0.001$).
