# Supplementary material for: The rice zebra3 (z3) mutation disrupts citrate distribution and produces transverse dark-green/green variegation in mature leaves
Source: Rice (N Y). 2018 Jan 5;11:1. doi: 10.1186/s12284-017-0196-8 (PMC5756232; doi:10.1186/s12284-017-0196-8)
Supplement: Supplementary file 3 — Amino acid alignment of Z3 homologs in higher plants. The amino acid sequences of Z3 homologs in higher plants were acquired from NCBI (http://www.ncbi.nlm.nih.gov/), and the amino acid alignment was obtained using the Clustal Omega EMBL-EBI (https://www.ebi.ac.uk/Tools/msa/clustalo/) and BoxShade 3.21 Server (https://www.ch.embnet.org/software/BOX_form.html). O. brachyantha, Z. mays_1, Z. mays_2, H. vulgare, S. bicolor, S. tuberosum, G. max, and A. thaliana have 99, 96, 95, 87, 72, 65, 64, and 62% sequence similarity to Z3, respectively. The mutated residue (S542P in z3) is indicated by a red arrowhead. O. sativa_ZEBRA3 (Oryza sativa ZEBRA3, LOC_Os03g05390, NP_001048962.1); O. brachyantha (Oryza brachyantha, XP_006649400.1); Z. mays_1 (Zea mays, NP_001151517.1); Z. mays_2 (Zea mays, ACG43196.1); H. vulgare (Hordeum vulgare, BAK05230.1); S. bicolor (Sorghum bicolor, XP_002467148.1); S. tuberosum (Solanum tuberosum, XP_006363328.1); G. max (Glycine max, XP_003533988.1); A. thaliana (Arabidopsis thaliana, NP_171728.2). (PDF 2499 kb) [file 12284_2017_196_MOESM3_ESM.pdf]

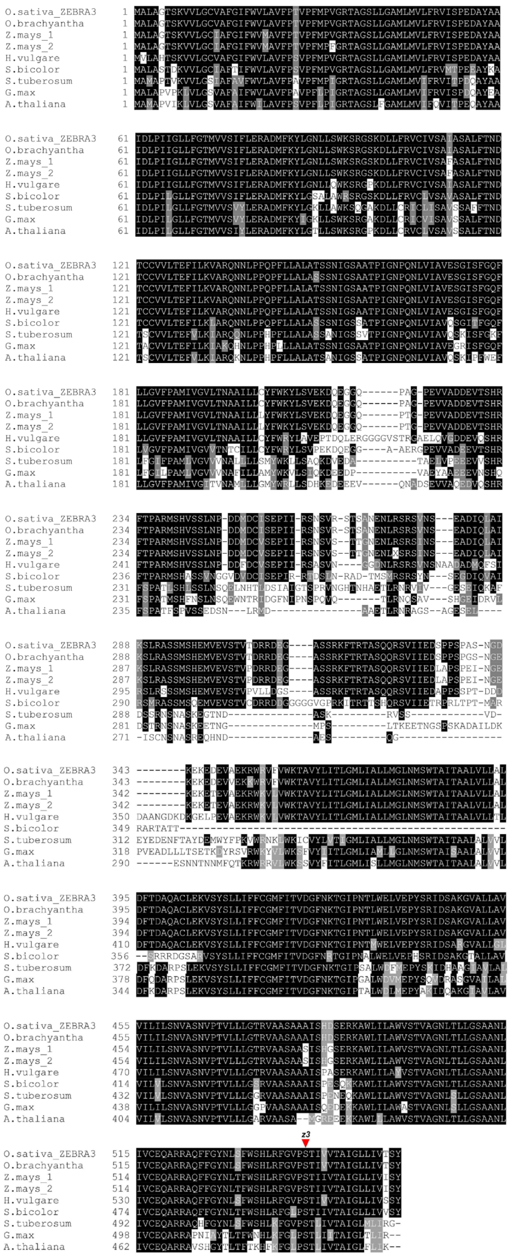

Additional File 3: Fig. S3 Amino acid alignment of Z3 homologs in higher plants.

The amino acid sequences of Z3 homologs in higher plants were acquired from NCBI (http://www.ncbi.nlm.nih.gov/), and the amino acid alignment was obtained using the Clustal Omega EMBL-EBI (http://www.ebi.ac.uk/Tools /msa/clustalo/) and BoxShade 3.21 Server (http://www.ch.embnet.org/soft ware/BOX_form.html). *O. brachyantha*, *Z. mays*_1, *Z. mays*_2, *H. vulgare*, *S. bicolor*, *S. tuberosum*, *G. max*, and *A. thaliana* have 99, 96, 95, 87, 72, 65, 64, and 62% sequence similarity to Z3, respectively. The mutated residue (S542P in *z3*) is indicated by a red arrowhead. *O. sativa_ZEBRA3* (*Oryza sativa* ZEBRA3, LOC_Os03g05390, NP_001048962.1); *O. brachyantha* (*Oryza brachyantha*, XP_006649400.1); *Z. mays*_1 (*Zea mays*, NP_001151517.1); *Z. mays*_2 (*Zea mays*, ACG43196.1); *H. vulgare* (*Hordeum vulgare*, BAK05230.1); *S. bicolor* (*Sorghum bicolor*, XP_002467148.1); *S. tuberosum* (*Solanum tuberosum*, XP_006363328.1); *G. max* (*Glycine max*, XP_003533988.1); *A. thaliana* (*Arabidopsis thaliana*, NP_171728.2).
