# Supplementary material for: The rice zebra3 (z3) mutation disrupts citrate distribution and produces transverse dark-green/green variegation in mature leaves
Source: Rice (N Y). 2018 Jan 5;11:1. doi: 10.1186/s12284-017-0196-8 (PMC5756232; doi:10.1186/s12284-017-0196-8)
Supplement: Supplementary file 4 — Confirmation of the overexpression of Z3 by the CaMV 35S promoter in six independent complementation lines. a Genomic PCR analysis for confirmation of rice transformation. The genomic region between the vector and the Z3 transgene was amplified in the transformed lines. All six independent lines were confirmed to be transformed. Primer information is listed in Additional file 8: Table S1. EV, empty vector; Control, vector used for transformation without the Z3 gene. b The overexpression of Z3 by the CaMV 35S promoter in six independent complementation lines. Primers were designed to amplify the transgene mRNA, and are listed in Additional file 8: Table S1. The GAPDH mRNA level was measured as a loading control. (PDF 647 kb) [file 12284_2017_196_MOESM4_ESM.pdf]

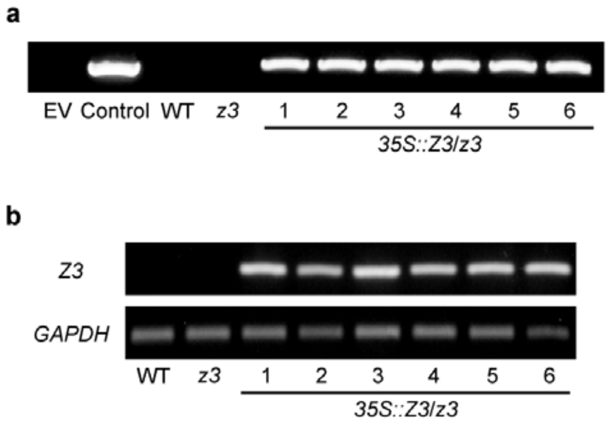

Additional File 4: Fig. S4 Confirmation of the overexpression of *Z3* by the CaMV 35S promoter in six independent complementation lines.

a Genomic PCR analysis for confirmation of rice transformation. The genomic region between the vector and the *Z3* transgene was amplified in the transformed lines. All six independent lines were confirmed to be transformed. Primer information is listed in **Additional file 8: Table S1**. EV, empty vector; Control, vector used for transformation without the *Z3* gene. **b** The overexpression of *Z3* by the CaMV 35S promoter in six independent complementation lines. Primers were designed to amplify the transgene mRNA, and are listed in **Additional file 8: Table S1**. The *GAPDH* mRNA level was measured as a loading control.
