# Supplementary material for: The rice zebra3 (z3) mutation disrupts citrate distribution and produces transverse dark-green/green variegation in mature leaves
Source: Rice (N Y). 2018 Jan 5;11:1. doi: 10.1186/s12284-017-0196-8 (PMC5756232; doi:10.1186/s12284-017-0196-8)
Supplement: Supplementary file 5 — Phenotypes of the homozygous T-DNA null mutants of Z3. a Schematic diagrams of the T-DNA insertion. The z3–2 and z3–3 mutants have a T-DNA insertion in the first exon, but in different locations. These T-DNA knockout mutants of Z3 were derived from the japonica rice cv ‘Hwayoung’. Filled boxes and thick lines indicate exons and introns, respectively. Primers LP1 (left primer 1), RP1 (right primer 1), LP2 (left primer 2), RP2 (right primer 2), and RB (right border primer) used for genotyping are represented as arrows and listed in Additional file 8: Table S1. b Phenotypes of leaf blades from four-month-old WT (Hwayoung), and z3–2 and z3–3 mutants grown under natural long day (NLD) conditions in the paddy field. c–d Panicle and grain phenotypes of the WT (Hwayoung) and two z3-knockout mutants at 150 DAS grown in a paddy field. e Days to heading data of the WT, and the z3–2, and z3–3 mutants in natural long-day conditions. Means and standard deviations were obtained from 5 plants of each genotype. Error bars indicate SD. Differences between means were compared using Student’s t-test (*** P < 0.001). f Genotyping of T-DNA insertion lines. Each LP and RP primer should have amplified about 1.1 kb of genomic DNA if no T-DNA insertion was made. If T-DNA was inserted, the length between the two primers was too large to be amplified. The RB and each RP primer should have amplified an approximately 0.6-kb band if T-DNA was inserted in the first exon. WT (Hwayoung) was used for the control. g Expression of Z3 in the WT and T-DNA knockout mutants by semi-quantitative RT-PCR. Z3 transcripts were not detected in the T-DNA knockout mutants. Ubiquitin 5 (UBQ5) mRNA was measured as a loading control. db, dull-brown colored grain; iv, ivory colored grain. (PDF 1621 kb) [file 12284_2017_196_MOESM5_ESM.pdf]

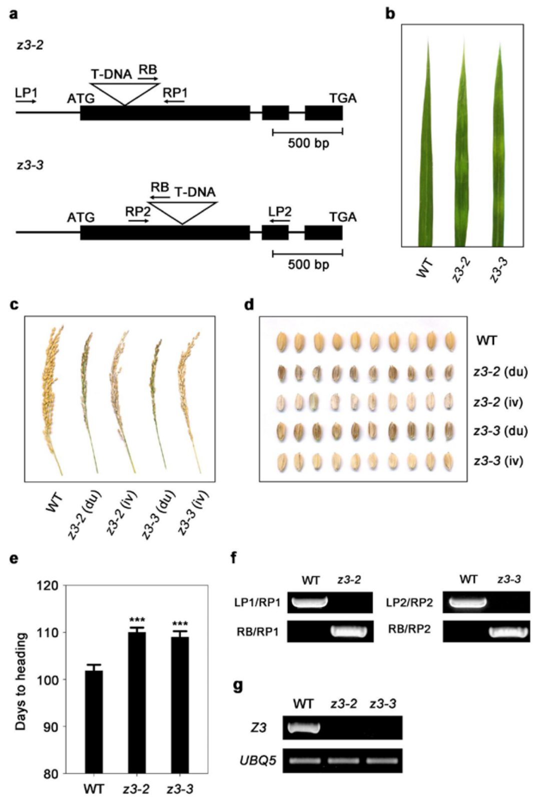

Additional File 5: Fig. S5 Phenotypes of the homozygous T-DNA null mutants of *Z3*.

a Schematic diagrams of the T-DNA insertion. The *z3-2* and *z3-3* mutants have a T-DNA insertion in the first exon, but in different locations. These T-DNA knockout mutants of *Z3* were derived from the *japonica* rice cv 'Hwayoung'. Filled boxes and thick lines indicate exons and introns, respectively. Primers LP1 (left primer 1), RP1 (right primer 1), LP2 (left primer 2), RP2 (right primer 2), and RB (right border primer) used for genotyping are represented as arrows and listed in Additional file 8: Table S1. **b** Phenotypes of leaf blades from four-month-old WT (Hwayoung), and *z3-2* and *z3-3* mutants grown under natural long day (NLD) conditions in the paddy field. **c–d** Panicle and grain phenotypes of the WT (Hwayoung) and two *z3*-knockout mutants at 150 DAS grown in a paddy field. **e** Days to heading data of the WT, and the *z3-2*, and *z3-3* mutants in natural long-day conditions. Means and standard deviations were obtained from 5 plants of each genotype. Error bars indicate SD. Differences between means were compared using Student's *t*-test (*** $P < 0.001$). **f** Genotyping of T-DNA insertion lines. Each LP and RP primer should have amplified about 1.1 kb of genomic DNA if no T-DNA insertion was made. If T-DNA was inserted, the length between the two primers was too large to be amplified. The RB and each RP primer should have amplified an approximately 0.6-kb band if T-DNA was inserted in the first exon. WT (Hwayoung) was used for the control. **g** Expression of *Z3* in the WT and T-DNA knockout mutants by semi-quantitative RT-PCR. *Z3* transcripts were not detected in the T-DNA knockout mutants. *Ubiquitin 5* (*UBQ5*) mRNA was measured as a loading control. db, dull-brown colored grain; iv, ivory colored grain.
