# Supplementary material for: The rice zebra3 (z3) mutation disrupts citrate distribution and produces transverse dark-green/green variegation in mature leaves
Source: Rice (N Y). 2018 Jan 5;11:1. doi: 10.1186/s12284-017-0196-8 (PMC5756232; doi:10.1186/s12284-017-0196-8)
Supplement: Supplementary file 6 — Analysis of reactive oxygen species (ROS) in the z3 mutant leaves. a-b Hydrogen peroxide (H2O2) and superoxide anion radicals (O2-) in flag leaves of the WT and z3 mutants at 160 DAS grown under natural long day conditions were visualized by staining with DAB (a) and NBT (b), respectively. Leaves before (left) and after (right) staining are shown. (PDF 497 kb) [file 12284_2017_196_MOESM6_ESM.pdf]

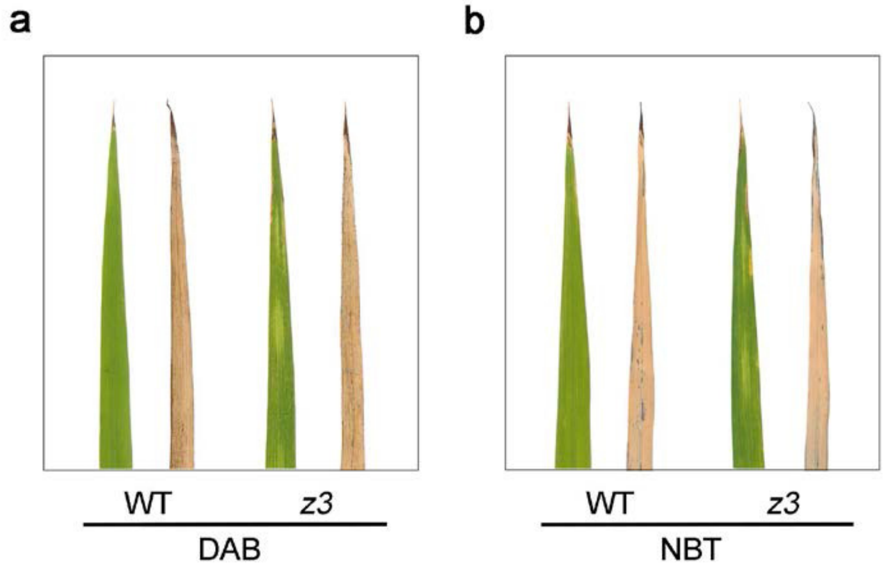

Additional File 6: Fig. S6 Analysis of reactive oxygen species (ROS) in the *z3* mutant leaves. **a-b** Hydrogen peroxide (H_2O_2) and superoxide anion radicals (O_2^-) in flag leaves of the WT and *z3* mutants at 160 DAS grown under natural long day conditions were visualized by staining with DAB (**a**) and NBT (**b**), respectively. Leaves before (left) and after (right) staining are shown.
