# Supplementary material for: The rice zebra3 (z3) mutation disrupts citrate distribution and produces transverse dark-green/green variegation in mature leaves
Source: Rice (N Y). 2018 Jan 5;11:1. doi: 10.1186/s12284-017-0196-8 (PMC5756232; doi:10.1186/s12284-017-0196-8)
Supplement: Supplementary file 7 — Transmission electron microscopy analysis of chloroplasts in the z3 mutant leaves. a-c Chloroplasts in the green leaves of the WT (a) and in the dark-green (b) and green (c) sectors of the z3 mutant leaves. Flag leaves of the 150-day-old WT and z3 mutant grown under natural long day conditions were sampled for analysis. G, grana thylakoid. Scale bars = 0.5 μm. (PDF 955 kb) [file 12284_2017_196_MOESM7_ESM.pdf]

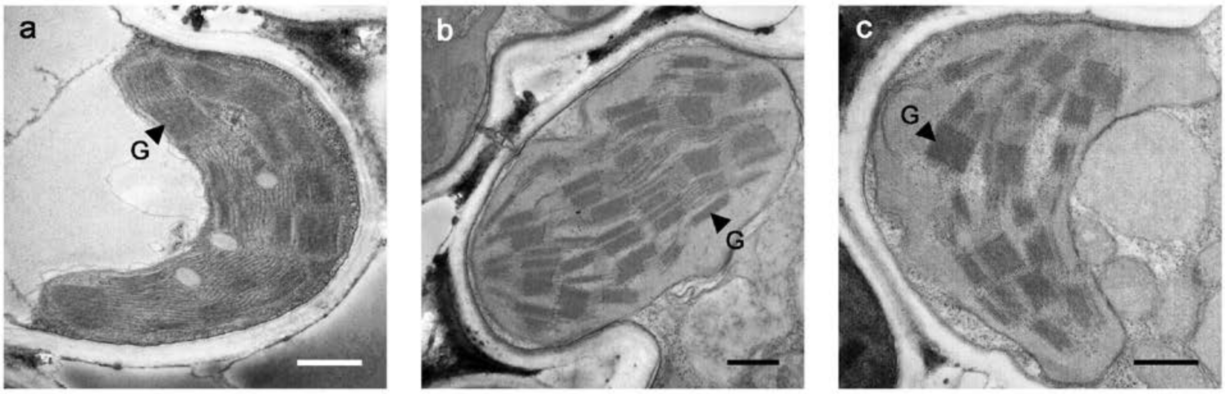

Additional File 7: Fig. S7 Transmission electron microscopy analysis of chloroplasts in the *z3* mutant leaves.

a-c Chloroplasts in the green leaves of the WT (**a**) and in the dark-green (**b**) and green (**c**) sectors of the *z3* mutant leaves. Flag leaves of the 150-day-old WT and *z3* mutant grown under natural long day conditions were sampled for analysis. G, grana thylakoid. Scale bars = 0.5 μm.
