# Supplementary material for: The rice zebra3 (z3) mutation disrupts citrate distribution and produces transverse dark-green/green variegation in mature leaves
Source: Rice (N Y). 2018 Jan 5;11:1. doi: 10.1186/s12284-017-0196-8 (PMC5756232; doi:10.1186/s12284-017-0196-8)
Supplement: Supplementary file 8 — Primers used in this study. (PDF 1453 kb) [file 12284_2017_196_MOESM8_ESM.pdf]

Additional File 8: Table S1. Primers used in this study.

I. The SSR, STS, and dCAPS markers used for map-based cloning of the *zebra3* locus		
Marker	Forward primer (5′→3′)	Reverse primer (5′→3′)
STS1	AGGGCATATATTTGTGTTAGTCCT	GTTGGCGTGGCAGTCCTAAC
dCAPS7	AGTGGCGGCTGTACAGAAATC	TCACCCACACAAGACACTGAGGGATC
dCAPS2	CTTGAGAGGTATGGAGAATGGTA	CAGCTCCTTTCGGCTAGCACTGCA
STS3	GAAGAGGTTGGCAGTTTGGC	GAGCTGAACTCGCTGTCAGT
RM14395	AATATCCGCAGCCGAAACATAGC	ACCGACCAAACCAACACAATCG
RM3126	TTCTTGCTCGTCTGCCTCC	CATCTTGCCATGCCTGATG
RM489	ACTTGAGACGATCGGACACC	TCACCCATGGATGTTGTCAG
RM5444	AACGTACCAGTCGCTGGTTC	CCCGTGATTTCCTCCGAC
RM7576	CTGCCCTGCCTTTTGTACAC	GCGAGCATTCTTTCTTCCAC
RM1022	CATGGGATGAGGGAGTAATG	CTTTGATAGCGGCTTTGTCC
RM1338	GAAGGGATTCAAGGGGTAGG	TCCAGTCATCCCTGTGAAAG
RM7	TTCGCCATGAAGTCTCTCG	CCTCCCATCATTTCGTTGTT
RM1284	ACACTCCCCTTTTGTAAAGC	CTAGAACTACGGCATTTTCG
RM5748	CAGTTGGCAATTGTCACGAG	TCGAACATATCCAAGCCTCC
RM16	CGCTAGGGCAGCATCTAAA	AACACAGCAGGTACGCGC
RM168	TGCTGCTTGCCTGCTTCCTTT	GAAACGAATCAATCCACGGC
RM3585	TTGATGAGAGTGAACCAGCG	CGTTTATGCAGAACAACCCC
II. Primer set used for cloning *Z3*		
Gene	Forward primer (5′→3′)	Reverse primer (5′→3′)
Z3	ATGGCGTTGGCAGGGACCTCC	GTAACTGGTGACGATGAGCA
III. Primer sets used for semi-quantitative RT-PCR or qRT-PCR		
Gene	Forward primer (5′→3′)	Reverse primer (5′→3′)
Z3 (Full)	ATGGCGTTGGCAGGGACCTCC	GTAACTGGTGACGATGAGCA
Z3 (Transgene-1)	AGGGTGTTGCTCTTCTTGCTGTG	TAATTTATCCTAGTTTGCGCGCT
Z3 (qRT-PCR)	AGGGTGTTGCTCTTCTTGCTGTG	CGAGCCCAGTAGTGTGAGGTTCC
Ubiquitin5	ACCACTTCGACCGCCACTACT	ACGCCTAAGCCTGCTGGTT
GAPDH	TTGGTGACAACAGGTCAAGC	AAACTTGTCGCTCAATGCAA
IV. Primer set used for confirmation of rice transformation		
Primer	Forward primer (5′→3′)	Reverse primer (5′→3′)
Z3 (Transgene-2)	CTATCCTTCGCAAGACCCTT	TGACGACCATGGTGCCAAAGA
V. Primer sets used for genotyping of T-DNA insertion lines		
Primer	Forward primer (5′→3′)	Reverse primer (5′→3′)
z3-2 (LP1/RP1)	CGTGCAGTCGATCTAACACC	ATCATCCGGGTTCAGAGATG
z3-2 (RB/RP1)	TTGGGGTTTCTACAGGACGTAAC	ATCATCCGGGTTCAGAGATG
z3-3 (LP2/RP2)	ACCTGTGGGAACATTTGAGG	TCTTCACCAATGACACCTGC
z3-3 (RB/RP2)	TTGGGGTTTCTACAGGACGTAAC	TCTTCACCAATGACACCTGC
VI. Primer set used for dCAPS analysis		
Primer	Forward primer (5′→3′)	Reverse primer (5′→3′)
dCAPS	CCACCTCCGGTTCGGGGTGAC	CAAAGGGTTCGTTCATCTATCTC
